# In Vitro Antibacterial Activity, Molecular Docking, and ADMET Analysis of Phytochemicals from Roots of *Dovyalis abyssinica*

**DOI:** 10.3390/molecules29235608

**Published:** 2024-11-27

**Authors:** Dereilo Bekere Belitibo, Asfaw Meressa, Abiy Abebe, Temesgen Negassa, Milkyas Endale, Frehiwot Teka Assamo, Messay Wolde-Mariam, Temesgen Abdisa Ayana, Marcel Frese, Norbert Sewald, Negera Abdissa

**Affiliations:** 1 Traditional and Modern Medicine Research and Development Directorate, Armauer Hansen Research Institute, Addis Ababa P.O. Box 1005, Ethiopia; derilobakere@gmail.com (D.B.B.); asfawmeresa03@gmail.com (A.M.); abiyabg@yahoo.com (A.A.); temesgen.negassa@gmail.com (T.N.); milkyasendale@yahoo.com (M.E.); frehiwot.teka@ahri.gov.et (F.T.A.); 2 Department of Chemistry, College of Natural and Computational Sciences, Wollega University, Nekemte P.O. Box 395, Ethiopia; 3 Pharmaceutical Industry Development Sector, Armauer Hansen Research Institute, Addis Ababa P.O. Box 1005, Ethiopia; messay.woldemariam@ahri.gov.et; 4 Department of Chemistry, College of Natural Sciences, Jimma University, Jimma P.O. Box 378, Ethiopia; temeabdi65@gmail.com; 5 Department of Chemistry, Organic and Bioorganic Chemistry, Bielefeld University, P.O. Box 100131, 33501 Bielefeld, Germany; marcel.frese@uni-bielefeld.de

**Keywords:** *Dovyalis abyssinica*, phytochemicals, antibacterial, ADMET, molecular docking

## Abstract

*Dovyalis abyssinica* is widely used in Ethiopia for treating various human ailments, yet its pharmacological properties and chemical composition remain largely unexplored. The chromatographic separation of *D. abyssinica* roots extract afforded five compounds, namely tremulacin (**1**), cochinchiside A (**2**), 5-methoxydurmillone (**3**), catechin-7-*O*-α-L-rhamnopyranoside (**4**), and stigmasterol (**5**), confirmed via IR, NMR, and MS spectral data. This is the first report of these compounds from this plant, except for compounds **1** and **5**. The extracts and isolated compounds were tested for antibacterial activity against *S. aureus*, *S. epidermidis*, *E. faecalis*, *E. coli*, *K. pneumoniae*, and *P. aeruginosa* strains. Methanol roots extract exhibited significant antibacterial activity (MIC 0.195 mg/mL) against *E. coli* and *P. aeruginosa*. Compounds **1** and **3** showed remarkable antibacterial activity, with compound **1** (MIC 0.625 mg/mL) exhibiting antibacterial activity against *S. aureus* and *S. epidermidis*, whereas compound **3** (MIC 0.625 mg/mL) exhibited antibacterial activity against *S. epidermidis* and *K. pneumoniae*. Molecular docking analysis revealed better binding energies for compound **1** (−8.0, −9.7, and −8.0 kJ/mol) and compound **3** (−9.0, −8.7, and −8.4 kJ/mol), compared to ciprofloxacin (−8.3, −7.5, and −6.7 kJ/mol), in regard to *S. aureus* pyruvate kinase, *S. epidermidis* FtsZ, and *K. pneumoniae* Topoisomerase IV, respectively. ADME analysis also revealed good antibacterial candidacy of these compounds, provided that in vivo analysis is conducted for further confirmation of the results.

## 1. Introduction

Infectious diseases remain the leading cause of deaths globally, could be due to the emergency of antimicrobial resistant pathogens [1]. This burden is particularly pronounced in developing countries, where the economic situations increase the prevalence of associated factors [2]. Despite the long history of using plants and plant-based products for treating diseases, such natural sources continue to be the source of antimicrobial chemicals [3]. With increasing antibiotic resistance and limited accessibility to pharmaceuticals in low-resource areas, medicinal plants offer promising, accessible, and culturally acceptable alternatives for infectious disease management. In fact, the World Health Organization (WHO) estimates that over 80% of the African population relies on traditional medicines for primary healthcare, due to their high availability, low cost, and the associated socio-cultural background [3]. However, these resources have been insufficiently studied from a scientific point of view, with most research limited to ethnomedicinal surveys. The isolation of antimicrobial drugs, including pencillins [4,5] and other β-lactams [6,7], from natural sources have been a significant milestone in the discovery of natural product-based medicines for combating infectious diseases.

*D. abyssinica* (family: Salicaceae), commonly known as the African gooseberry, is native to Africa [8] and has long been used in traditional medicine for treating various human ailments. The roots are used for the treatment of stomachaches, fevers [9], reproductive health conditions [10], epilepsy [11], and rheumatic pain [12], while the stem bark has been used for the treatment of cancer [13,14] and ascaris [12]. The leaves are used to treat body swelling [15], blood pressure, asthma [16], and colds [17]. The fruits are used to treat abdominal pain [18,19] and cancer [20]. The seeds are used to treat hemorrhoids [15]. Despite its extensive use in ethnomedicine, limited scientific studies exist on the medicinal properties of *D. abyssinica*. Therefore, the current study aims to investigate the chemical constituents of root extract, evaluate their antibacterial activities, in silico molecular docking study and ADMET predictions to assess the potential of the isolated compounds as drug candidates, bridging traditional uses with modern pharmacological insights.

## 2. Results and Discussion

### 2.1. Secondary Metabolites Isolated from the Roots of D. abyssinica

**Figure 1:**
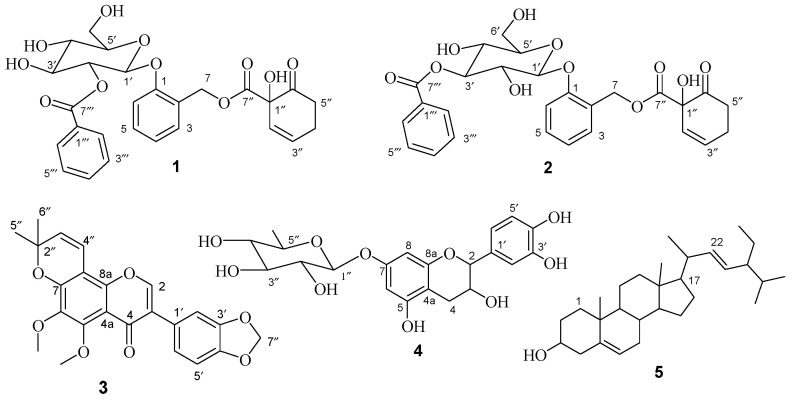
The structures of the isolated compounds (**1**–**5**).

The chromatographic separation of *D. abyssinica* roots extract yielded five compounds (**1**–**5**; Figure 1), with compounds **2**–**4** reported for the first time from this species. The structures of the compounds were determined using IR, NMR (1D and 2D), and MS spectroscopic techniques, as detailed below. The data were also compared with the values in the literature to confirm the structure of the isolated compounds.

**Figure 2:**
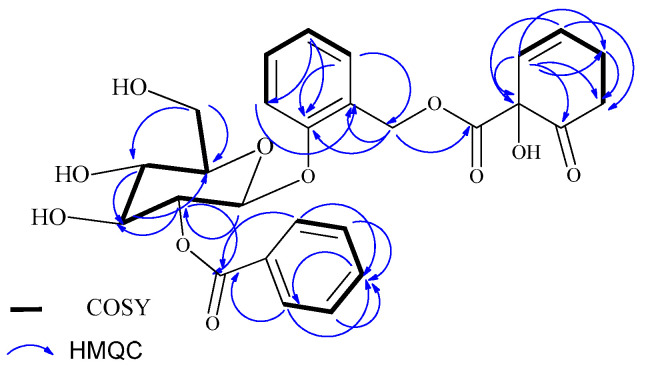
Key ^1^H-^1^H COSY (bold line) and HMBC (curved arrow) correlations of compound **1**.

Compound **1** was isolated as a white powder and its molecular formula, C_27_H_28_O_11_, was deduced from the ESI-MS analysis, with a molecular ion peak at *m*/*z* 551 ([M+Na]^+^) for the sodium adduct and the ^13^C NMR data. The ^1^H NMR spectrum (500 MHz, acetone-d_6_, Table 1 and Supplementary Figure S1) showed three mutually coupled aromatic protons with an A_2_B_2_C spin pattern, centered at δ_H_ 8.08 (d, *J* = 8.3 Hz, 2H), 7.51 (t, *J* = 7.8 Hz, 2H), and 7.64 (t, *J* = 7.4 Hz, 1H), that were assigned to H-2‴/H-6‴, H-3‴/H-5‴, and H-4‴, respectively, in terms of the mono-substituted benzoyl moiety. The aromatic proton signals at δ_H_ 7.28 (d, *J* = 8.4 Hz, 1H), 7.02 (d, *J* = 7.4 Hz, 1H), 7.25 (d, *J* = 7.8 Hz, 1H), and 7.24 (d, *J* = 7.8 Hz, 1H), indicated the presence of a 1, 2-disubstituted aromatic ring. The doublet proton signals at δ_H_ 5.75 (d, *J* = 9.8 Hz, 1H) and δ_H_ 6.13 (m) indicated the presence of olefinic protons and were assigned to H-2″ and H-3″, respectively. The presence of a phenolic glycoside was also evidenced by the presence of an aliphatic doublet at δ_H_ 5.37 (d, *J* = 8.0 Hz, 1H) and multiplets at δ_H_ 5.31 (m, 1H), 3.97 (m, 1H), 3.69 (m, 1H), 3.69 (m, 1H), 4.00–3.91 (m, 1H),and 3.87–3.76 (m, 1H), showing HSQC correlations with carbons at δ_C_ 99.4 (C-1′), 74.3 (C-2′), 77.8 (C-3′), 70.6 (C-4′), 74.38 (C-5′), and 61.5 (C-6′), respectively. The ^13^C NMR spectrum (Table 1 and Supplementary Figure S2) showed 27 well-resolved carbon signals, corresponding to 7quaternary, 16methine, and 4 methylene carbons. The downfield signal at δ_C_ 205.4 revealed the presence of a carbonyl moiety. The carbon signals resonating at δ_C_ 165.2 and 169.7 indicated the presence of two ester groups. The HMBC spectrum showed long-range correlations in terms of proton at H-7 (δ_H_ 4.97) with the carbon at C-7″ (δ_C_ 169.7) and H-2′ (δ_H_ 5.31) with C-7‴ (δ_C_ 165.2), which confirm the placement of the two ester moieties at C-7 and C-2′, respectively. Further comprehensive analyses of the 2D NMR (^1^H-^1^H COSY, ^1^H-^13^C HMQC, and long-range HMBC; Figure 2) experiments (Supplementary Figures S4–S6) and a comparison with the data from the literature [21,22,23,24] revealed that the structure of compound **1** was tremulacin, previously reported from the root part of the same plant [21].

**Figure 3:**
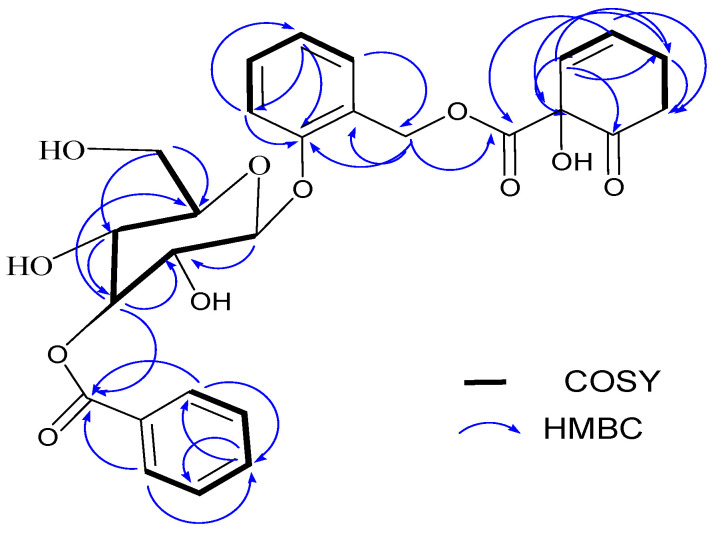
Key ^1^H-^1^H COSY (bold line) and HMBC (curved arrow) correlations of compound **2**.

Compound **2** was also isolated as a light yellow solid. The IR (KBr disk) spectrum (Supplementary Figure S15) showed broad absorption band at 3343 cm^−1^, sharp absorption at 1621 cm^−1^, 1600 cm^−1^, and 1255 cm^−1^ attributed to hydroxyl moiety (OH), C=O, C=C, and C-O stretching vibrations, respectively. The strong absorption band at 2925 cm^−1^ showed the presence of C-H stretching of the sp^3^ aliphatic moiety. The absorption band at 1750 cm^−1^ showed the presence of C=O stretching of the ester moiety. It exhibited similar spectral properties to compound **1**, including the molecular ion in the ESI-MS spectrum at *m*/*z* 551 for [M+Na]^+^ and 27 carbon signals in the ^13^C NMR spectrum (Table 1 and Supplementary Figure S9). The only difference is for the downfield chemical shift value for H-3′ (δ_H_ 5.33), compared to that of compound **1** (H-3′; δ_H_ 3.97) (Table 1). This indicated that the benzoate ester group is at the C-3′ position in the glucose unit in compound **2**. This argument is further supported by long-range HMBC correlations (Figure 3), indicating correlations between proton H-3′ (δ_H_ 5.33) and the benzoate carbonyl carbon C-7‴ (δ_C_ 166.0), suggesting that the benzoyl moiety is attached to the C-3′ position. Based on the aforementioned information and comprehensive analyses of the 2D NMR spectrum (^1^H-^1^H COSY and HMBC) and a comparison with reported data, the structure of compound **2** was identified as cochinchiside A [25]. It is worth mentioning that both compounds **1** and **2** have been co-isolated from the root bark of *Homalium cochinchinensis* [25].

Compound **3** was isolated as a white solid. The IR spectrum exhibited a strong absorption band for conjugated carbonyl at 1641 cm^−1^. In addition, the medium absorption bands, around 1600 cm^−1^ and 1470 cm^−1^, suggest the presence of an aromatic system in the compound. The molecular formula, C_23_H_20_O_7_, was determined from the ESI-MS analysis (*m*/*z* 431 for ([M+Na]^+^) and ^13^C NMR data, indicating the presence of signals for 23 carbon atoms. The ^1^H and ^13^C NMR spectra (Table 1 and Supplementary Figures S16–S19) revealed the presence of a typical isoflavone, as evidenced by the ^1^H and ^13^C NMR spectra showing singlets at δ_H_ 7.83 for H-2 and δ_C_ 150.3 for C-2, respectively. The ^1^H NMR spectrum further showed an AB spin system consisting of two doublets at δ_H_ 5.70 and 6.76 ppm (*J* = 10.0 Hz), which, together with the 6H singlet at δ_H_ 1.27 ppm, suggested the presence of a 2″,2″-dimethylpyrano moiety. In the aromatic region, the ABX spin system appeared at δ_H_ 7.09 (1H, d, *J* = 2.4 Hz), 6.87 (1H, d, *J* = 8.0 Hz), 6.94 ppm (1H, dd, *J* = 2.4 and 7.8 Hz), and together with the two-proton singlet at δ_H_ 5.99 ppm for the oxymethylene group. The presence of twothree-proton singlets at δ_H_ 3.97 and 3.91 ppm for two methoxy groups was also evident. The ^13^C NMR spectrum (Table 1) showed twenty-three carbon signals, which accounted for one carbonyl, six sp^2^methines, two methyl, two methoxy, ten sp^2^ quaternary, and one sp^3^ quaternary carbon atoms. These data are consistent with the compound being 5-methoxydurmillone [26,27], which was previously reported in the root parts of *Millettia ferruginea* [26].

Compound **4** was isolated as a light brown solid. The characteristic proton signals in the ^1^H NMR (Table 1 and Supplementary Figure S20) spectrum at δ_H_ 4.49, 3.83, 2.68, and 2.36, for H-2, H-3, H-4b, and H-4a, respectively, along with two aromatic spin systems with AB (at δ_H_ 5.90 and 6.26) and ABX (at δ_H_ 6.90, 6.68, and 6.60) patterns clearly indicated the catechin skeleton of the compound [28]. A doublet signal at δ_H_ 4.80 (d, *J* = 52.2 Hz, 1H) and multiplet signals around δ_H_ 3.40–4.02 indicated the presence of a sugar moiety [29,30]. The ^13^C NMR spectrum (Table 1) displayed carbon signals that correspond to a total of 21 carbon atoms, with the characteristics of 12 aromatic carbons and 9 non-aromatic carbons, including the anomeric carbon. The linkage of this sugar was established at C-7, following the HMBC correlation of the anomeric protons, H-1,″ with the oxygenated aromatic quaternary carbon C-7. Thus, based on the analysis of these spectral data and a comparison with the data reported in the literature [28,29,30], the structure of compound **4** was identified as catechin-7-*O*-α-L-rhamnopyranoside. This is the first report of its isolation from this plant, having been previously reported from the root of *Ulmus davidiana* [28].

Compound **5** was isolated as a white powder. The FT-IR spectrum (Supplementary Figure S26) showed a broad band at 3413 cm^−1^, suggesting the presence of an O-H stretching vibration in an alcohol group. The bands observed at 2935 and 2860 cm^−1^ showed the asymmetrical and symmetrical C-H stretching vibrations of sp^3^ hydrocarbons. The bands displayed at 1464 and 1383.16 cm^−1^ represent the C-H bending vibrations of methyl groups. Weak bands, observed at 1650 and 1048 cm^−1^, were assigned to C=C and C-O stretching vibrations, respectively. The ^1^H NMR spectrum (Supplementary Figure S23) displayed three doublet of doublets at δ_H_ 5.35 (dd, *J* = 5.3, 1.9 Hz, 1H), 5.17 (dd, *J* = 15.2, 8.6 Hz, 1H), and 5.04 (dd, *J* = 15.2, 8.7 Hz, 1H), which are typical signals for the olefinic proton (H-6) of the steroidal skeleton and olefinic protons (H-22 and H-23), respectively. The proton signals at δ_H_ 3.54 (dd, *J* = 11.1, 4.6 Hz, 1H), integrating for 1H, were indicative of the hydroxymethine proton, H-3. Two singlet peaks at δ_H_ 0.70 (s, 3H) and 1.01 (s, 3H) were assigned to two tertiary methyl groups attached to C-13 and C-10, respectively. The spectrum further revealed three doublet peaks at δ_H_ 1.04 (3H), 0.84 (3H), and 0.86 (3H), which were assigned to the methyl groups attached at C-20, C-25, and C-25, respectively. The ^13^C NMR spectrum (Supplementary Figure S24) also showed the presence of 29 carbon atoms, of which a hydroxyl carbon signal at δ_C_ 72.0 is assignable to C-3. The signals at δ_C_ 140.9, 121.9, 138.5, and 129.4 correspond to the olefinic carbons at C-5, C-6, C-22, and C-23, respectively. Based on these data and a comparison with the data reported in the literature [31,32,33], the structure of compound **5** (Figure 1) was found to be stigmasterol.

### 2.2. Antibacterial Activity

By using the microdilution method, the antibacterial activity (Table 2) of the extract and the isolated compounds were examined against three Gram-positive (*S. aureus*, *S. epidermidis*, and *E. faecalis*) and three Gram-negative (*E. coli*, *K. pneumoniae*, and *P. aeruginosa*) bacterial strains (Table 2 and Supplementary Figures S27 and S28). These strains are among the drug-resistant pathogenic bacteria applicable to most first-line drugs [34,35] and any active substance to these strains is also believed to be active on other bacterial strains. Each microorganism was examined with a serial bi-fold dilution, from a high concentration to a low concentration, for both the extract and isolated compounds, to determine the MIC values. Both the extract and isolated compounds demonstrated antibacterial activity, with varying degrees of responses against the bacterial strains. The crude extract showed considerable antibacterial activity against the bacterial strains, with a minimum inhibition concentration (MIC) ranging from 0.195 to 6.250 mg/mL. The highest level of antibacterial activity, with an MIC value of 0.195 mg/mL, was observed against both *E. coli* and *P. aeruginosa*. A related study found that a crude methanol extract and a fraction of a leaf from *D. abyssinica* displayed significant antibacterial activity against *S. aureus*, *E. faecalis*, and *E. coli* [36].

The study revealed that the isolated compounds exhibited varying levels of growth inhibition across different bacterial strains, underscoring both subtle and significant differences in the effectiveness of their antibacterial activities. This variability suggests that each compound interacts uniquely with the biological mechanisms of the specific strains, likely due to genetic or metabolic differences influenced by the structural characteristics of the compounds.

Compounds **1** and **3** exhibited the strongest antibacterial activities, with compound **1** showing an MIC value of 0.625 mg/mL against both *S. aureus* and *S. epidermidis.* Similarly, compound **3** displayed equipotent efficacy against *S. epidermidis* and *K. pneumoniae*, with MIC values of 0.625 mg/mL for both bacterial strains, highlighting its potential as an effective antibacterial agent. A related study found that compound **1** was also effective against the same pathogens, significantly inhibiting microbial growth [24]. The potency of compound **1** and **3** suggests that they may share similar mechanisms or target sites, indicating a significant affinity for bacterial pathways and likely disrupting essential growth processes. In contrast, compounds **2**, **4**, and **5** exhibited medium-to-low potency against the tested bacterial strains. A related study demonstrated that compound **5** inhibited *S. typhimurium* [33]. Compound **2**, which has a structure that is closely related to compound **1**, differing only in the position of the benzoate ester group at C-3′ of the glucose unit, displayed weaker activity against the tested bacterial strains (MIC value ≥ 5.00 mg/mL).

The variation in the antibacterial activities of compounds **1** and **2** with closer structural feature could be due to the position of the benzoate ester group and/or their combined effect. The position of the benzoate ester group within the molecular structure, along with its stereochemical structure, could play a crucial role in how effectively these compounds interact with bacterial targets.

There is also an observed difference in sensitivity based on the Gram stain (+ve or −ve), as clearly demonstrated by compound **1**, which showed a higher level of growth inhibition in terms of the Gram-positive bacteria, with MIC values of 0.625, 0.625, and 1.25 mg/mL, against *S. aureus*, *S. epidermidis*, and *E. faecalis*, respectively, in contrast to the Gram-negative bacteria, *E. coli*, *K. pneumoniae*, and *P. aeruginosa*, with MIC values of ≥5.00 mg/mL for these strains. In some cases, the crude extracts appeared to have even better antibacterial activity in comparison to the isolated compounds. The better antibacterial activity of the crude extract could be linked to the synergistic interactions of several secondary metabolites present in the extract, which may not be the case when single compounds are evaluated alone [37]. In general, the results on the antibacterial activity of the crude extract and isolated compounds are in line with the traditional utilization of this plant, clearly indicating the antibacterial potential of this medicinal plant as a good source for lead compounds in the development of antibacterial drugs.

### 2.3. Molecular Docking Studies on the Isolated Compounds

**Figure 4:**
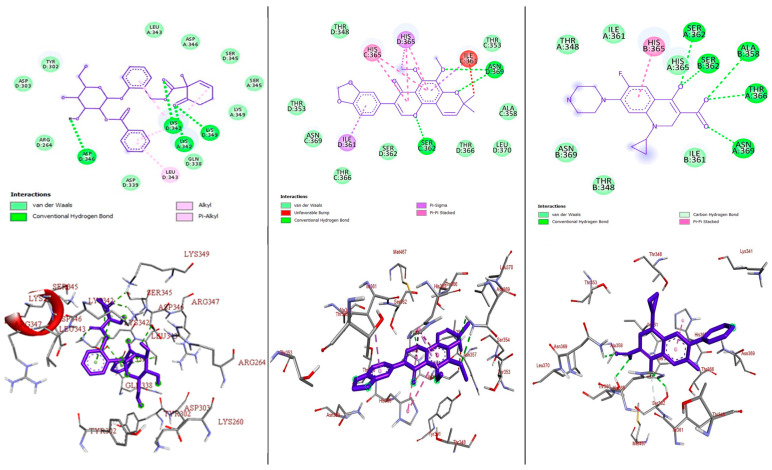
The 2D (**top**) and 3D (**bottom**) binding interactions of compound **1**, **3**, and ciprofloxacin (from **left** to **right**) against *S. aureus* pyruvate kinase (PDB ID: 3T07).

**Figure 5:**
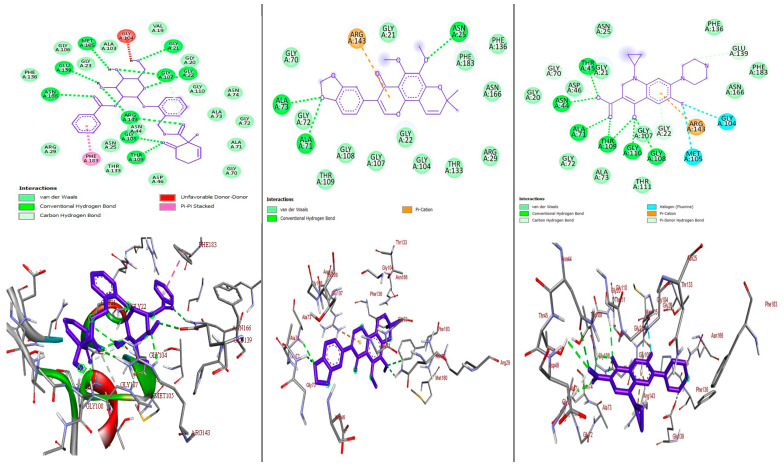
The 2D (**top**) and 3D (**bottom**) binding interactions of compound **1**, **3**, and ciprofloxacin against *S. epidermidis* FtsZ (PDB ID: 4M8I).

**Figure 6:**
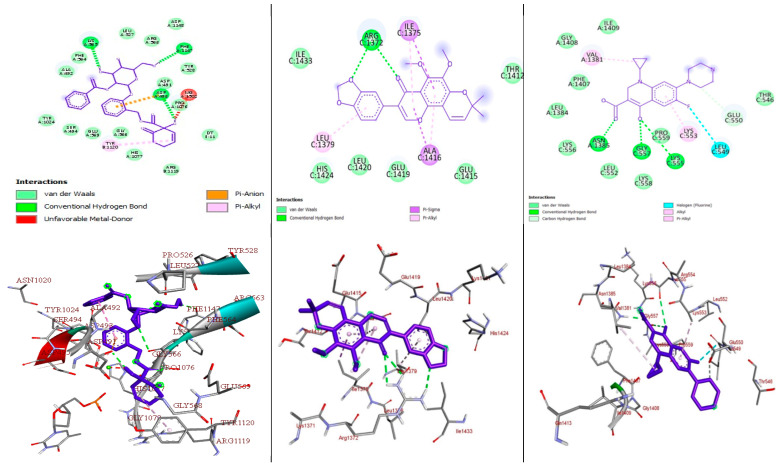
The 2D (**top**) and 3D (**bottom**) binding interactions of compound **1**, **3**, and ciprofloxacin against *K. pneumoniae* topoisomerase IV (ParE–ParC) in complex with DNA (PDB ID: 7LHZ).

Most pharmaceuticals and biologically active compounds exert their effects by interacting with specific protein targets, making target identification crucial in drug development and biomedical research, particularly since many drugs can interact with multiple proteins. In this study, we conducted molecular docking analyses to assess the binding affinity and interactions of the most active compounds, **1** and **3**, against the target proteins, namely pyruvate kinase of *S. aureus* (PDB ID: 3T07), FtsZ of *S. epidermidis* (PDB ID: 4M8I), and topoisomerase IV (ParE–ParC) of *K. pneumoniae* in complex with DNA (PDB ID: 7LHZ), utilizing protein crystal structures from the Protein Data Bank (PDB). Using the AutoDock Vina platform, we determined that a more negative binding energy value indicates a better binding affinity between the ligand and the target proteins. The efficacy of most antibiotics relies on their ability to selectively target critical proteins that are essential for bacterial survival; for instance, pyruvate kinase in *S. aureus* catalyzes the final rate-limiting step in glycolysis, crucial for carbohydrate metabolism [38]. Similarly, FtsZ in *S. epidermidis* is vital for cell division and the pharmacological inhibition of this protein, by targeting its inter-domain binding site, can lead to the development of drugs that disrupt its interaction with nucleotides, thus inhibiting cell division [39]. Furthermore, topoisomerase IV in *K. pneumoniae*, composed of ParE and ParC, is pivotal for DNA replication, repair, and maintenance, by regulating the DNA’s topological structure, making it another promising target for drug design [40]. We examined the binding affinity, interactions, and amino acid residues of compounds **1** and **3**, along with ciprofloxacin, a gyrase inhibitor that disrupts bacterial DNA replication [41]. Ciprofloxacin also shows potential as a secondary target when docked to pyruvate kinase, which is crucial for glycolysis and energy production [42,43,44], and has been reported to bind to FtsZ, impacting bacterial cell division [45]. Positive results from the experiments with standard ciprofloxacin guided our exploration of this docking approach, summarized in Figure 4, Figure 5 and Figure 6 and Supplementary Tables S1–S3.

#### 2.3.1. Molecular Docking Analysis of Compounds **1** and **3** Against *S. aureus* Pyruvate Kinase (PDB ID: 3T07)

Pyruvate kinase is an enzyme present at the final stage of glycolysis that catalyzes the transfer of the phosphoryl group from phosphoenolpyruvate (PEP) to adenosine diphosphate (ADP) and generates adenosine triphosphate (ATP) and pyruvate [42]. This reaction is a major control point in the regulation of glycolytic flux [43]. It also plays a key role in controlling metabolic flux distribution [44]. Inhibiting this enzyme disrupts the energy production and metabolic stability of bacteria. The binding affinities of isolated compounds **1** and **3** were determined by docking towards pyruvate kinase in the *S. aureus* (PDB ID: 3T07) receptor. The results were compared to ciprofloxacin (Figure 4, Supplementary Table S1). The minimum binding energies of these compounds in the binding pocket of pyruvate kinase in *S. aureus* were −8.0 and −9.0 kJ/mol, respectively, the results of which are better than for the reference drug, ciprofloxacin (−8.3 kJ/mol). The high binding affinity of these compounds against pyruvate kinase in *S. aureus* could be due to the presence of hydroxyl and other functional groups involved in hydrogen bonding, hydrophobic interactions, and van der Waals interactions with amino acid residues to form a ligand–protein complex. The antibacterial activity values are in good agreement with the molecular docking results for the compounds.

#### 2.3.2. Molecular Docking Analysis of Compounds **1** and **3** Docked Against *S. epidermidis* FtsZ (PDB ID: 4M8I)

*S. epidermidis* FtsZ (PDB ID: 4M8I) is a crucial protein involved in bacterial cell division and the molecules that bind to it are thought to disrupt its connection with nucleotides, thereby inhibiting cell division. The binding affinities of compounds **1** and **3** were assessed against *S. epidermidis* FtsZ and compared to ciprofloxacin, which is known to interact with FtsZ and affect its function during the cell division process (Figure 5, Supplementary Table S2). The compounds showed binding energies of −9.7 and −8.7 kcal/mol, respectively, the results of which is better than for the standard drug, ciprofloxacin (−8.0 kcal/mol), indicating that these compounds could be antibacterial agents. The binding affinities of these compounds were stabilized through hydrogen bonding, the hydrophobic interaction, and the van der Waals interaction with amino acid residues, as indicated in Supplementary Table S2 and Figure 5.

#### 2.3.3. Molecular Docking Analysis of Compounds **1** and **3** Docked Against *K. pneumoniae* Topoisomerase IV (ParE–ParC) in Complex with DNA (PDB ID: 7LHZ)

In this study, compounds **1** and **3** were docked against *K. pneumoniae* topoisomerase IV (ParE–ParC) in complex with DNA (PDB ID: 7LHZ) and ciprofloxacin, as a reference control. The results demonstrated that both compounds **1** (−8.0 kcal/mol) and **3** (−8.4 kcal/mol) have a better docking affinity within the binding pocket of *K. pneumoniae* topoisomerase IV (ParE–ParC) in complex with DNA than the standard drug, ciprofloxacin (−6.7 kcal/mol). The compounds showed at least one hydrogen bonding interaction within the active site of *K. pneumoniae* topoisomerase IV (ParE–ParC) in complex with DNA with key amino acid residues. The binding affinity, hydrogen bonding, hydrophobic interaction, and van der Waals interaction of the compounds are summarized in Figure 6 and Supplementary Table S3.

The 2D and 3D binding interactions of the compounds and ciprofloxacin against *K. pneumoniae* topoisomerase IV (ParE–ParC) in complex with DNA are depicted in Figure 6. The ribbon model shows the binding pocket structure of *K. pneumoniae* topoisomerase IV (ParE–ParC) in complex with DNA with compounds **1**, **3**, and ciprofloxacin. The hydrogen bonds between the compounds and amino acids are shown as green dashed lines and the hydrophobic interactions are represented by pink lines.

### 2.4. In Silico Pharmacokinetics (Drug-Likeness Properties) and Toxicity Analysis

The structures of the isolated compounds (**1** and **3**) were converted into their canonical simplified molecular input-line entry system (SMILE) and imported in to the Swiss ADME tool to estimate the in silico pharmacokinetic parameters (drug-likeness properties), according to Lipinski’s rule of five [46,47] and Veber’s rule [48]. Lipinski’s rule of five implies that the drugs and/or drug candidates should obey the five-parameter rule, which states that hydrogen-bond donors (NHDs) should be less than 5, hydrogen-bond acceptors (NHAs) should be less than 10, the molecular mass should be less than (MW) 500 Daltons, and the log P should be less than 5. In regard to Veber’s rule, a compound with <10 rotatable bonds (RTBs) and a polar surface area (TPSA) of <140 Å^2^ should present good oral bioavailability. A compound’s drug-likeness is a prediction that screens whether a particular organic molecule has properties consistent with being an orally active drug [46,47].

The Swiss ADME prediction (Table 3) showed that the compounds (**1** and **3**) satisfied Lipinski’s rule of five, except for compound **1**, which showed two violations due to the high number of hydrogen acceptors (NHAs > 10) and the molecular mass (MW > 500 Daltons). The ADME analysis of compound **1**, previously isolated from *Solanum dasyphyllum* roots, is consistent with the present study [24]. The molecular weight of compound **3** was found to be <500 Daltons, indicating easy absorption, diffusion, and transportation. The topological polar surface area (TPSA) value was recorded as 76.36 Å^2^ and was well below the limit of 140 Å^2^. The calculated number of rotatable bond (NRB) values was less than 10, which indicated that compound **3** was conformationally stable. Compound **3** with fewer or, preferably, no violations is likely to be considered as a potential drug candidate.

The skin permeability value (Kp) in cm/s indicates the skin absorption of molecules. The skin permeability, Kp, value of compound **1** was −8.32 cm/s and compound **3** was −6.05 cm/s, indicating moderate skin permeability and being within the range of the broad-spectrum antibiotic, ciprofloxacin (−9.09 cm/s). Additionally, gastrointestinal (GI) and blood–brain barrier (BBB) permeation indicate the absorption and distribution of drug molecules. The Swiss ADME prediction parameters indicated that compound **3** had high gastrointestinal (GI) absorption, whereas tremulacin (**1**) displayed low absorption. In the same way, both compounds **1** and **3** showed good blood brain barrier (BBB) permeation. Moreover, a range of cytochromes (CYPs) regulate drug metabolism, in which CYP1A2, CYP2C19, CYP2C9, CYP2D6, and CYP3A4 are vital for the biotransformation of drug molecules. Thus, compound **3** inhibited all cytochromes, except CYP1A2 and CYP2D6 and the substrate of the permeability glycoprotein (P-gp) (Table 4).

The toxicity profiles of the compounds were created using ProTox-II and OSIRIS Property Explorer. An acute toxicity prediction indicated that none of the isolated compounds (**1** and **3**) had acute toxicity (LD_50_ >5000, toxicity class > 5). The ProTox-II toxicological prediction provides results for endpoints such as hepatotoxicity, carcinogenicity, immunotoxicity, mutagenicity, and cytotoxicity. Both compounds **1** and **3** were predicted to be non-carcinogenic, non-irritant, and inactive against hepatotoxicity, mutagenicity, and cytotoxicity. The toxicity profile of compound **1**, isolated from the roots of *Solanum dasyphyllum*, is also in good agreement with the present study [24]. The results of the PreADMET and OSIRIS Property Explorer prediction analyses are shown in Table 5.

## 3. Materials and Methods

### 3.1. General Experimental Procedures

All the solvents and reagents used for extraction and purification were analytical and HPLC grade. The TLC analysis was performed using analytical TLC pre-coated sheets, ALUGRAM^®^ Xtra SIL G/UV_254_ (layer: 0.20 mm, silica gel 60, with fluorescent indicator UVF254/365). Silica gel, with a mesh size of 100–200, was used for the column chromatography. The chromatograms were visualized on TLC by spraying them with 10% H_2_SO_4_ and heating them on a hot plate. The NMR spectra data were recorded using an Advance 500 MHz spectrometer (Bruker, Billerica, MA, USA). The chemical shifts (δ) were measured in parts per million (ppm) and referenced to the resonance peak of the appropriate deuterated solvent (residual CDCl_3_ at δ_H_ 7.26 for protons and δ_C_ 77.0 for carbons) downfield of trimethylsilane (TMS), which served as the internal reference. For the antimicrobial analysis, Whatman No. 1 filter paper (Whatman Ltd. International, Maidstone, UK), Tween, Petri dishes, pre-labeled 96-well microplates, a biosafety cabinet (Telstar), a UV–Visible spectrophotometer (Thermo Scientific Evolution 60S), an incubator (Memmert, Germany), a whirl mixer (borfax), and Mueller–Hinton broth (MHB) (Sigma-Aldrich, Darmstadt, Germany), were used, and ciprofloxacin (Barleben, Germany, Lot: R12590) was used as the reference standard.

### 3.2. Plant Material Collection and Identification

The roots of *D. abyssinica* were collected from around Shonie town, east Badawachoworada, Hadiya zone, the central Ethiopia region, in December 2021. The plant material was identified by a botanist (Prof. Sileshi Nemomisa) and the voucher specimen (DB-05) has been deposited in Addis Ababa University’s Herbarium.

### 3.3. Extraction and Isolation

The air-dried roots of *D. abyssinica* (1 kg) were first defatted with *n*-hexane (3 × 4 L) in Erlenmeyer flasks for 24 h through maceration, while shaking, using an electronic shaker at a speed of 150 rpm, at room temperature. After 24 h, the solution was filtered using Whatman No.1 filter paper. The residue was dried and then soaked with methanol (4 L), using the same procedure as mentioned above. The filtered solution was dried using a rotary evaporator, at a temperature of 40 °C, to produce 57.3 g of crude extract.

About 50.0 g of the crude extract was suspended in water and then partitioned successively with *n*-hexane, chloroform, and ethyl acetate, to provide 6 g, 24 g, and 15 g extracts, respectively. The chloroform and ethyl acetate extracts were combined together, based on their TLC profiles. The combined extract (35 g) was subjected to silica gel (250 g) column chromatography (CC) and the column was eluted with an increasing polarity of EtOAc in *n*-hexane in order to provide 185 fractions, 40 mL each. Fractions 54–70, eluted using *n*-hexane/EtOAc (9:1), were combined and re-chromatographed, with isocratic *n*-hexane/EtOAc (9:1) as the eluent, resulting in compounds **3** (12 mg) and **5** (24 mg). Fractions 90–120, eluted with *n*-hexane/EtOAc (7:3), showed two closer spots on the TLC. These fractions were combined and further purified by using column chromatography isocratic *n*-hexane/EtOAc (7:3) as an eluent, followed by Sephadex LH-20 (eluent: dichloromethane/methanol, 1:1) gel filtration, resulting in compound **1** (25 mg) and **2** (21 mg). Fractions 122–135, eluted with *n*-hexane/EtOAc (6:4), were also combined and further purified using column chromatography (an increasing gradient of EtOAc in *n*-hexane) and Sephadex LH-20 (eluted with dichloromethane/methanol (1:1), resulting in compound **4** (15 mg).

### 3.4. Pathogenic Bacterial Strains

Three Gram-positive (*Staphylococcus aureus* (ATCC 25923), *Staphylococcus epidermidis* (ATCC 12228), and *Enterococcus faecalis* (ATCC 29212) and three Gram-negative (*Escherichia coli* (ATCC 25922), *Klebsiella pneumoniae* (ATCC 700603), and *Pseudomonas aeruginosa* (ATCC 27853) bacterial strains were used to evaluate the activity of the extract and isolated compounds. The strains were obtained from the Ethiopian Public Health Institute (EPHI) and were maintained in triptosoya + 20% glycerol broth, at −78 °C, in the microbiology laboratory of the Traditional and Modern Medicine Research and Development Directorate (TMMRDD) at the Armauer Hansen Research Institute (AHRI).

### 3.5. Inoculum Preparation

The inoculum size of the tested bacteria was standardized, according to the Clinical and Laboratory Standards Institute’s guidelines [49,50]. From stored stock cultures, the bacteria were refreshed for the actual tests in Petri dishes containing nutrient agar, by incubating them for 18–24 h at 37 °C. The refreshed test organisms were standardized by diluting them with broth and measuring their absorbance, using a spectrophotometer adjusted to 625 nm, with an absorbance reading OD value range from 0.08 to 0.1, which is equivalent to 1.0 × 10^8^ CFU/mL bacteria species. The suspensions were diluted in broth to get the final inoculum size of 5 × 10^5^ CFU/mL [51,52].

### 3.6. Evaluation of the Antibacterial Activity

The antibacterial activity of the methanol extract and isolated compounds were tested against six pathogenic bacteria (three Gram positive and three Gram negative), using broth micro-dilutions (minimum inhibitory concentration) for the MIC assays, as described in the literature [53,54,55,56].

#### 3.6.1. Minimum Inhibitory Concentration

All the tested extracts/compounds were manipulated to determine their minimum inhibitory concentration (MIC) using the micro-broth dilution method, by preparing different concentrations of the extracts and isolated compounds. Then, the prepared concentrations of the extracts and isolated compounds were incorporated into 96-well microplates, with 100 µL of the broth medium and thoroughly mixed 8–10 times, with the broth serially diluted two-fold (25 mg/mL to 0.195 mg/mL for extracts) in order to achieve the (5 mg/mL to 0.078 mg/mL for pure compounds) test concentration, followed by the inoculation of a 100 µL of the defined bacterial inoculum suspensions (5 × 10^5^ CFU/mL). The microplates were incubated in suitable conditions at 37 °C for 18–24 h. Then, bacterial growth was examined after 18–24 h of incubation. After incubation, 40 µL of 0.4 mg/mL 2,3,5-triphenyltetrazolium chloride (TTC) solution was added to each 96-well microplate and re-incubated for 30 min at 37 °C to confirm the presence or absence of purple color formation, which is an indicator of microbial growth. The MIC values were determined visually through observation with magnifying instruments. All the assays were performed in triplicate [57]. Different quality controls were performed parallel to the experimental tests to assess the accuracy of the test. In order to determine the susceptibility of the bacteria to the tests, positive control experiments with ciprofloxacin were conducted in parallel at a starting concentration of 10 μg/mL. Additionally, negative controls, sterility controls, and growth controls were run parallel to the experimental tests. The MIC value was considered the lowest concentration that inhibited the growth of the respective bacteria under suitable incubation conditions and was expressed in mg/mL.

#### 3.6.2. Data Analysis

The experiments were performed in triplicate and the outcomes were calculated as the mean value. Visual readings were taken of the results. The minimum inhibitory concentration (MIC) endpoint was defined as the lowest concentration of the antibacterial substance that prevented the overall growth of the microorganisms. The MIC value was determined by the presence or absence of a pink color, using magnifying instruments.

### 3.7. InSilico Molecular Docking Methodology

The interaction and binding affinity of the isolated compounds were investigated against *S. aureus* pyruvate kinase (PDB ID: 3T07), *S. epidermidis* FtsZ (PDB ID: 4M8I), and *K. pneumonia* topoisomerase IV (ParE–ParC) in complex with DNA (PDB ID: 7LHZ) proteins, which were downloaded from the Protein Data Bank. Protein assembly and docking were simulated using AutoDock Vina 4.2.6, using the energy-minimized ligand as the input. The protein was prepared using the standard protocol [58], which included removing the co-crystallized ligand, as well as selected water molecules and cofactors. The target protein file was prepared by leaving the associated residue with the protein, by using the auto-preparation of the target protein. The docking calculation results included the binding energy (kcal/mol), hydrogen bond distances, and a pictorial representation of viable docked poses. ChemDraw 16.0 was used to analyze each of the 2D structures of the isolated compounds. ChemBio3D was utilized to minimize the energy of every molecule. A maximum of nine conformers were considered for each ligand. The conformations with the most favorable (least) free-binding energy were selected for analysis of the interactions between the target receptor and ligands, using the Discovery Studio Visualizer. The grid box was constructed using a docking simulation, with measurements of 30 × 30 × 30 Å, pointing in x, y, and z directions, respectively, with a grid point spacing of 0.375 Å. The center grid box dimensions in the XYZ directions were 0.013907, 0.147852, and−0.003148 for 3T07; −18.825964, −9.619607, and 19.510250 for 4M8I; and 102.601077, −41.730449, and 110.767237 for 7LHZ.

### 3.8. InSilico Drug-Likeness Properties and Toxicity Predictions

The drug-likeness properties of the isolated compounds were predicted based on an already established protocol [46,47]. The structures of the isolated compounds were converted into the canonical simplified molecularinput-lineentry system (SMILE). They were imported into the SwissADME and PreADMET tools to estimate the pharmacokinetics [59,60]. The organ toxicities and toxicological endpoints of the isolated compounds were predicted using the PreADMET, ProTox-II, and OSIRIS properties [61,62].

## 4. Conclusions

The chromatographic separation of *D. abyssinica* root extracts led to the isolation of five compounds (**1**–**5**), namely tremulacin (**1**), cochinchiside A (**2**), 5-methoxydurmillone (**3**), catechin-7-*O*-α -L-rhamnopyranoside (**4**), and stigmasterol (**5**). Of these, compounds **2**, **3**, and **4** are reported herein for the first time from the species, while compounds **1** and **5** have been previously reported from this species. The structures of the isolated compounds were elucidated using IR, NMR (1D and 2D), and MS spectroscopic techniques. The extracts and isolated compounds displayed a broad range of antibacterial activity against the tested bacterial strains, with tremulacin (**1**) and 5-methoxydurmillone (**3**) displaying significant activities against *S. aureus*, *S. epidermidis*, and *K. pneumoniae,* comparable to ciprofloxacin. These results were in good agreement with or consistent with those of in silico molecular docking. These compounds showed better binding affinity against pyruvate kinase in *S. aureus*, *S. epidermidis* FtsZ, and *K. pneumoniae* topoisomerase IV in complex with DNA compared to the standard drug, ciprofloxacin, indicating that these compounds could be potential antibacterial agents. Furthermore, ADMET (drug-likeness) studies revealed that the compounds have drug-like characteristics and have the potential to be antibacterial lead compounds, provided that other extensive in vitro and in vivo evaluations are performed.

## Figures and Tables

**Table 1:** ^1^H NMR (600 MHz) and ^13^C NMR (150 MHz) spectral data of compounds **1**–**4** (in CDCl_3_), δ in ppm.

No	Compound 1		Compound 2		Compound 3		Compound 4	
	δ_H_ (m, *J* in Hz)	δ_C_	δ_H_ (m, *J* in Hz)	δ_C_	δ_H_ (m, *J* in Hz)	δ_C_	δ_H_ (m, *J* in Hz)	δ_C_
1		155.0		155.0				
2		125.0		125.0	7.83 (s)	150.3	4.49 (m)	81.5
3	7.28 (d, 8.4)	129.5	7.26 (t, 7.9)	130.1		125.4	3.83 (m)	66.8
4	7.02 (dd, 7.4, 1.3)	122.4	7.01 (t, 7.5)	123.1		175.1	2.68 (m), 2.36 (m)	29.5
4a						106.7		99.5
5	7.25 (d, 7.8)	128.3	7.17 (d, 7.5)	129.6		151.3		156.6
6	7.24 (d, 7.8)	115.4	7.05 (d, 8.2)	115.6		140.0	5.90 (s)	94.3
7	4.97 (s), 5.12 (m)	62.3	4.95 (d, 12.5), 5.09 (d, 12.5)	63.7		149.1		155.8
8						113.2	6.26 (s)	95.6
8a						153.4		157.0
1′	5.37 (d, 8.0)	99.4	5.16 (d, 7.8)	99.5		125.7		131.1
2′	5.31 (m)	74.3	3.94 (d, 4.9)	74.8	7.09 (d, 2.4)	110.0	6.90 (s)	115.0
3′	3.97 (m)	74.8	5.33 (d, 8.1)	74.0		147.6		145.3
4′	3.69 (m)	70.6	3.94 (m)	69.8		147.6		144.9
5′	3.69 (m)	77.3	3.55 (dt, 6.8)	76.1	6.87 (d 8.0)	108.3	6.68 (m)	115.5
6′	4.00–3.91 (m), 3.87–3.76 (m)	61.5	3.91 (t, 4.9)	61.3	6.94 (dd, 2.4, 7.8)	122.5	6.60 (m)	118.9
1″		77.9		78.4			4.80 (d, 52)	106.2
2″	5.76 (d, 9.8)	128.5	5.71 (d, 9.8)	127.5		78.2	4.02–3.40 (m)	78.5
3″	6.13 (m)	131.5	6.04 (dt, 9.8, 3.9)	132.1	5.70 (d, 10.0)	115.0	4.02–3.40 (m)	81.5
4″	2.54 (m)	26.3	2.44 (m), 2.5 (m)	26.7	6.76 (d, 10.0)	129.1	4.02–3.40 (m)	68.4
5″	2.86 (m)	35.2	2.52 (m), 2.96 (d, 15.4)	35.3	1.27 (s)	28.1	3.93 (d, 76.7)	65.4
6″		205.4		206.1	1.27 (s)	28.1	1.22 (d, 13.3)	16.4
7″		169.7		169.6	5.99 (s)	101.1		
1‴		130.3		129.5				
2‴	8.08 (d, 8.3)	129.6	8.02 (d, 7.7)	129.9				
3‴	7.51 (t, 7.8)	128.5	7.36 (t, 7.6)	128.4				
4‴	7.64 (t, 7.4)	133.3	7.51 (m)	133.3				
5‴	7.51 (t, 7.8)	128.5	7.36 (t, 7.6)	128.4				
6‴	8.08 (d, 8.3)	129.6	8.02 (d, 7.7)	129.9				
7‴		165.2		166.0				
5-OMe					3.97 (s)	62.2		
6-OMe					3.91 (s)	61.5		

**Table 2:** MIC values of the crude extract and isolated compounds (in mg/mL) against the tested microorganisms.

Bacterial Strains	Extract	Average MIC Values from Triplicate Assays of Compounds and Reference Drug					
		1	2	3	4	5	Cipro
*S. aureus*	3.125	0.625	5.00	1.25	2.50	5.00	0.312
*S. epidermidis*	0.781	0.625	5.00	0.625	5.00	2.50	0.156
*E. faecalis*	6.25	1.25	5.00	1.25	5.00	2.50	0.312
*E. coli*	0.195	5.00	>5.00	5.00	2.50	5.00	0.078
*K. pneumoniae*	3.125	>5.00	>5.00	0.625	>5.00	>5.00	0.195
*P. aeruginosa*	0.195	5.00	>5.00	5.00	5.00	>5.00	<0.039

Key: Cipro = ciprofloxacin. Each value represents the mean value.

**Table 3:** Drug-likeness predictions for the compounds, computed by Swiss ADME.

Ligands	Mol. Wt. (g/mol)	NHD	NHA	NRB	TPSA (A^°2^)	Lipophilicity		Log *S* (ESOL) Water Solubility	Lipinski’s Rule of Five with Zero Violations
						Log *P* (iLOGP)	Log *P* (MLOGP)		
**1** (C_27_H_28_O_11_)	528.5	4	11	10	128.94	3.93	−0.06	−3.76	2
**3** (C_23_H_20_O_7_)	408.40	0	7	3	76.36	4.00	1.62	−5.00	0
Ciprofloxacin (C_17_H_18_FN_3_O_3_)	331.34	2	5	3	74.57	2.24	1.28	−1.32	0

Key: NHD = number of hydrogen donors, NHA = number of hydrogen acceptors, NRB = number of rotatable bonds, and TPSA = total polar surface area.

**Table 4:** ADME predictions for the compounds, computed by Swiss ADME and PreADMET.

Ligands	(Log *Kp*) cm/s	GIA	BBB	Inhibitor Interaction					
				P-gp	CYP1A2 Inhibitor	CYP2C19 Inhibitor	CYP2C9 Inhibitor	CYP2D6 Inhibitor	CYP3A4 Inhibitor
**1** (C_27_H_28_O_11_)	−8.32	Low	No	Yes	No	No	No	No	No
**3** (C_23_H_20_O_7_)	−6.05	High	No	Yes	No	Yes	Yes	No	Yes
Ciprofloxacin (C_17_H_18_FN_3_O_3_)	−9.09	High	No	Yes	No	No	No	No	No

Key: BBB = blood–brain barrier permeability; CYP = cytochrome-P; GIA = gastrointestinal absorption; LogKp = skin permeation value; P-gp = P-glycoprotein substrate.

**Table 5:** Toxicity prediction for the compounds, computed by ProTox-II and OSIRIS Property Explorer.

Ligands	LD_50_ (mg/kg)	Toxicity Class	Organ Toxicity					
			Hepato	Carcino	Immuno	Mutagen	Cytoto	Irritant
**1** (C_27_H_28_O_11_)	5000	5	Inactive	Inactive	Active	Inactive	Inactive	No
**3** (C_23_H_20_O_7_)	3850	5	Inactive	Inactive	Active	Inactive	Inactive	No
Ciprofloxacin (C_17_H_18_FN_3_O_3_)	2000	4	Inactive	Inactive	Inactive	Active	Inactive	No

Key: Hepato = hepatotoxicity; Carcino = carcinogenicity; Immuno = immunotoxicity; Mutagen = mutagenicity; Cytoto = cytotoxicity; LD = lethal dose.

## Data Availability

NMR data for compounds **1**–**5**, as well as the photographs of the antibacterial activity, using the minimum inhibitory concentrations of the extracts and isolated compounds, are included in the Supplementary Materials.

## Supplementary Materials

The following supporting information can be downloaded at: https://www.mdpi.com/article/10.3390/molecules29235608/s1, Figure S1: ^1^H NMR spectrum of DBB-20/Tremulacin (**1**); Figure S2: The ^13^C NMR spectrum of DBB-20/Tremulacin (**1**); Figure S3: DEPT-135 spectrum of DBB-20/Tremulacin (**1**); Figure S4: COSY spectrum of-DBB-20/Tremulacin (**1**); Figure S5: HMQC spectrum of DBB-20/Tremulacin (**1**); Figure S6: HMBC spectrum of DBB-20/Tremulacin (**1**); Figure S7: ESI-MS measurement or results of DBB-20/Tremulacin (**1**); Figure S8: ^1^H NMR spectrum DBB-23/Cochinchiside A (**2**); Figure S9: ^13^C NMR spectrum of DBB-23/Cochinchiside A (2); Figure S10: DEPT-135 spectrum of DBB-23/Cochinchiside A (**2**); Figure S11: COSY spectrum of-DBB-23/Cochinchiside A (**2**); Figure S12: HMQC spectrum of DBB-23 Cochinchiside A (**2**); Figure S13: HMBC spectrum of DBB-23 Cochinchiside A (2); Figure S14: ESI-MS measurement or results of DBB-23/Cochinchiside A (**2**); Figure S15: FT-IR spectrum of DBB-23/Cochinchiside A (2); Figure S16: The H NMR spectrum of DBB-36/5-methoxy durmillone (**3**); Figure S17: ^13^C NMR spectrum of DBB-36/5-methoxy durmillone (**3**); Figure S18: DEPT-135 spectrum of DBB-36/5-methoxy durmillone (**3**); Figure S19: ESI-MS measurement or results of DBB-36/5-methoxy durmillone (**3**); Figure S20: ^1^H NMR spectrum of DBB-34/catechin-7-O-α -L-rhamnopyranoside (**4**); Figure S21: ^13^C NMR spectrum of DBB-34 catechin-7-O-α -L-rhamnopyranoside (**4**); Figure S22: DEPT-135 spectrum of DBB-34/Catechin-7-O-α -L-rhamnopyranoside (**4**); Figure S23: ^1^H NMR spectrum of DBB-18/Stigmasterol (**5**); Figure S24: The ^13^C NMR spectrum of DBB-18/Stigmasterol(**5**); Figure S25: DEPT-135 spectrum of DBB -18/Stigmasterol (**5**); Figure S26: FT-IR spectrum of DBB-18/Stigmasterol (5); Table S1: Molecular docking results of compounds **1**, **3**, and ciprofloxacin against Pyruvate kinase of *S. aureus* (PDB ID 3T07); Table S2: Molecular docking results of compounds **1**, **3** and ciprofloxacin against *S. epidermidis* FtsZ (PDB ID 4M8I); Table S3: Molecular docking results of compounds **1**, **3** and ciprofloxacin against *K. pneumoniae* Topoisomerase IV (ParE-ParC) in complex with DNA (PDB ID 7LHZ); Figure S27: Photo of antibacterial activity by using the micro-broth dilution method MIC values of the crude extracts against tested microorganisms; Figure S28: Photo of antibacterial activity by using the micro-broth dilution method MIC values of the isolated compounds against tested microorganisms.
