# Supplementary material for: In Vitro Antibacterial Activity, Molecular Docking, and ADMET Analysis of Phytochemicals from Roots of Dovyalis abyssinica
Source: Molecules. 2024 Nov 27;29(23):5608. doi: 10.3390/molecules29235608 (PMC11644002; doi:10.3390/molecules29235608)
Supplement: Supplementary file 1 [file molecules-29-05608-s001.zip › molecules-3274946-supplementary.pdf]

Supplementary Materials

In vitro antibacterial activity, molecular docking, and ADMET analysis of phytochemicals from roots of *Dovyalis abyssinica*

Dereilo Bekere Belitibo[1,2], Asfaw Meressa[1], Abiy Abebe[1], Temesgen Negassa[1], Milkyas Endale[1], Frehiwot Teka Assamo[1], Messay Wolde-Mariam [3], Temesgen Abdisa Ayana[4], Marcel Frese[5], Norbert Sewald[5*], and Negera Abdissa[1,2,5*]

[1] Traditional and Modern Medicine Research and Development Directorate, Armauer Hansen Research Institute, P.O. Box 1005, Addis Ababa, Ethiopia; derilobakere@gmail.com (D.B.B.); asfawmeresa03@gmail.com (A.M.); abiyabg@yahoo.com (A.A.); temesgen.negassa@gmail.com (T.N.); milkyasendale@yahoo.com (M.E.); frehiwot.teka@ahri.gov.et (F.T.A.)

[2] Department of Chemistry, College of Natural and Computational Sciences, Wollega University, P.O. Box 395, Nekemte, Ethiopia

[3] Pharmaceutical Industry Development Sector, Armauer Hansen Research Institute, P.O. Box 1005, Addis Ababa , Ethiopia; messay.woldemariam@ahri.gov.et

[4] Department of Chemistry, College of Natural Sciences, Jimma University, Jimma, P.O. Box 378, Ethiopia; temeabdi65@gmail.com

[5] Department of Chemistry, Organic and Bioorganic Chemistry, Bielefeld University, P.O. Box 100131, 33501 Bielefeld, Germany; marcel.frese@uni-bielefeld.de

* Correspondences: norbert.sewald@uni-bielefeld.de (N.S.); negera.abdissa@uni-bielefeld.de (N.A.); Tel.: +49-521-106-2051 (N.S.); Tel.: +251-913-354-086 (N.A.)

Table of Contents

Spectroscopic data for Compound 1 2
Figure S1. ^{1}H NMR spectrum (500 MHz, acetone-d6) of DBB-20/ Tremulacin (1) 2
Figure S2. ^{13}C NMR spectrum(125 MHz, acetone-d6) of DBB-20/ Tremulacin (1) 3
Figure S3. DEPT-135 spectrum (125 MHz, acetone-d6) of DBB-20/ Tremulacin (1) 3
Figure S4. COSY spectrum (500 MHz, acetone-d6) of-DBB-20 / Tremulacin (1) 4
Figure S5. HMQC spectrum(500 MHz, 125 MHz acetone-d6) of DBB-20 / Tremulacin (1) 4
Figure S6. HMBC spectrum (500 MHz, 125 MHz acetone-d6) of DBB-20 / Tremulacin (1) 5
Figure S7: ESI-MS measurement or results of DBB-20/ Tremulacin (1) 5
Spectroscopic data for Compound 2 6
Figure S8. ^{1}H NMR spectrum (500 MHz, chloroform-d) DBB-23/ Cochinchiside A (2). 6
Figure S9. ^{13}C NMR spectrum(125 MHz, chloroform-d) of DBB-23/ Cochinchiside A (2). 6
Figure S10. DEPT-135 spectrum (125 MHz, chloroform-d) of DBB-23/ Cochinchiside A (2) 7
Figure S11. COSY spectrum (500 MHz, chloroform-d) of-DBB-23/ Cochinchiside A (2) 7
Figure S12. HMQC spectrum (500 MHz, 125 MHz chloroform-d) of DBB-23 Cochinchiside A (2) 8
Figure S13. HMBC spectrum (500 MHz, 125 MHz chloroform-d) of DBB-23 Cochinchiside A (2) 8
Figure S14: ESI-MS measurement or results of DBB-23/ Cochinchiside A (2) 9
Figure S15: FT-IR spectrum of DBB-23/ Cochinchiside A (2) 9
Spectroscopic data for Compound 3 10
Figure S16. ^{1}H NMR spectrum (400 MHz, chloroform-d) of DBB-36/5-methoxy durmillone (3) 10
Figure S17. ^{13}C NMR spectrum (125 MHz, chloroform-d) of DBB-36/ 5-methoxy durmillone (3) 10
Figure S18 . DEPT-135 spectrum (125 MHz, chloroform-d) of DBB-36/ 5-methoxy durmillone (3) 11
Figure S19: ESI-MS measurement or results of DBB-36/ 5-methoxy durmillone (3) 11
Spectroscopic data for Compound 4 12
Figure S20. ^{1}H NMR spectrum(400 MHz, Dmso) of DBB-34/catechin-7-O-α -L-rhamnopyranoside (4). 12

Figure S21. ^{13}**C NMR spectrum (125 MHz, Dmso) of DBB-34 catechin-7-O-α -L-rhamnopyranoside (4)12**
Figure S22. DEPT-135 spectrum (125 MHz, Dmso) of DBB-34/ Catechin-7-O-α -L-rhamnopyranoside (4)13
Spectroscopic data for Compound 5.. 13
Figure S23. ^{1}**H NMR spectrum(500 MHz, chloroform-d) of DBB-18/Stigmasterol(5) 13**
Figure S24. ^{13}**C NMR spectrum(125 MHz, chloroform-d) of DBB -18/Stigmasterol(5)............................. 14**
Figure S25. DEPT-135 spectrum(125 MHz, chloroform-d) of DBB -18/Stigmasterol (5) 14
Figure S26: FT-IR spectrum of DBB -18/Stigmasterol (5) 15
Table S1. Molecular docking results of compounds 1, 3, and ciprofloxacin against Pyruvate kinase of S. aureus (PDB ID: 3T07) (Binding Affinity in kcal/mol) 15
Table S2. Molecular docking results of compounds 1, 3, and ciprofloxacin against S. epidermidis FtsZ (PDB ID: 4M8I) (Binding Affinity in kcal/mol) 16
Table S3. Molecular docking results of compounds 1, 3 and ciprofloxacin against K. pneumoniae Topoisomerase IV (ParE-ParC) in complex with DNA (PDB ID: 7LHZ) (Binding Affinity in kcal/mol)....... 16
Information/photo of Antibacterial activity.. 17
Figure S27. Photo of Antibacterial activity by using the micro-broth dilution method MIC values of the crude extracts against tested microorganisms 17
Figure S28. Photo of Antibacterial activity by using the micro-broth dilution method MIC values of the isolated compounds against tested microorganisms.. 18

Spectroscopic data for Compound 1

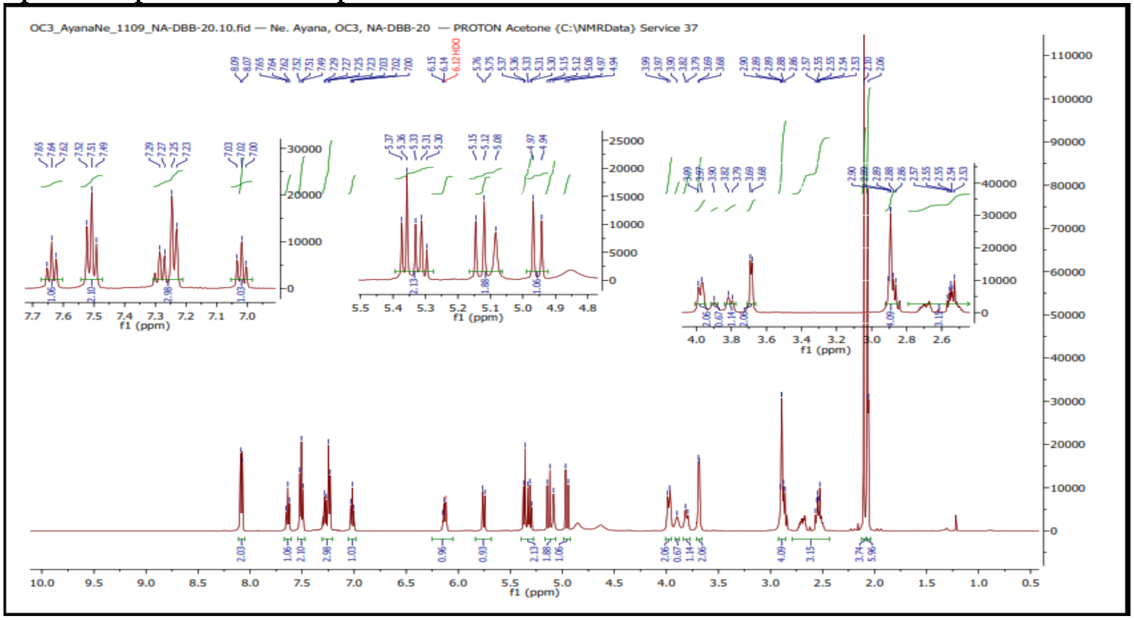

Figure S1. ^{1}H NMR spectrum (500 MHz, acetone-*d*6) of DBB-20/ Tremulacin (1)

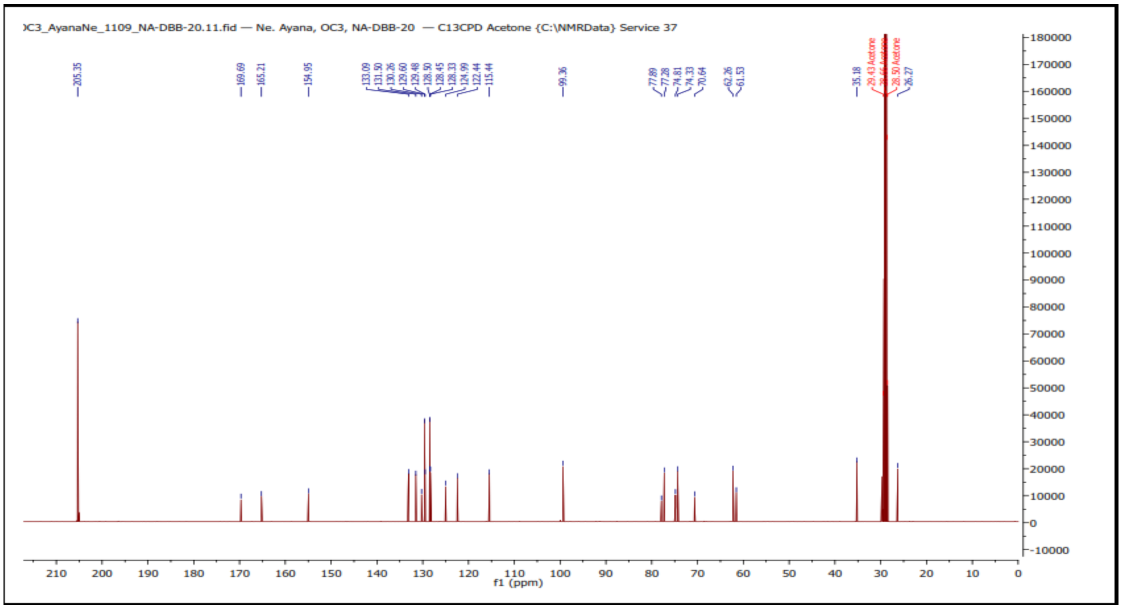

Figure S2. ^{13}C NMR spectrum(125 MHz, acetone-*d*6) of DBB-20/ Tremulacin (**1**)

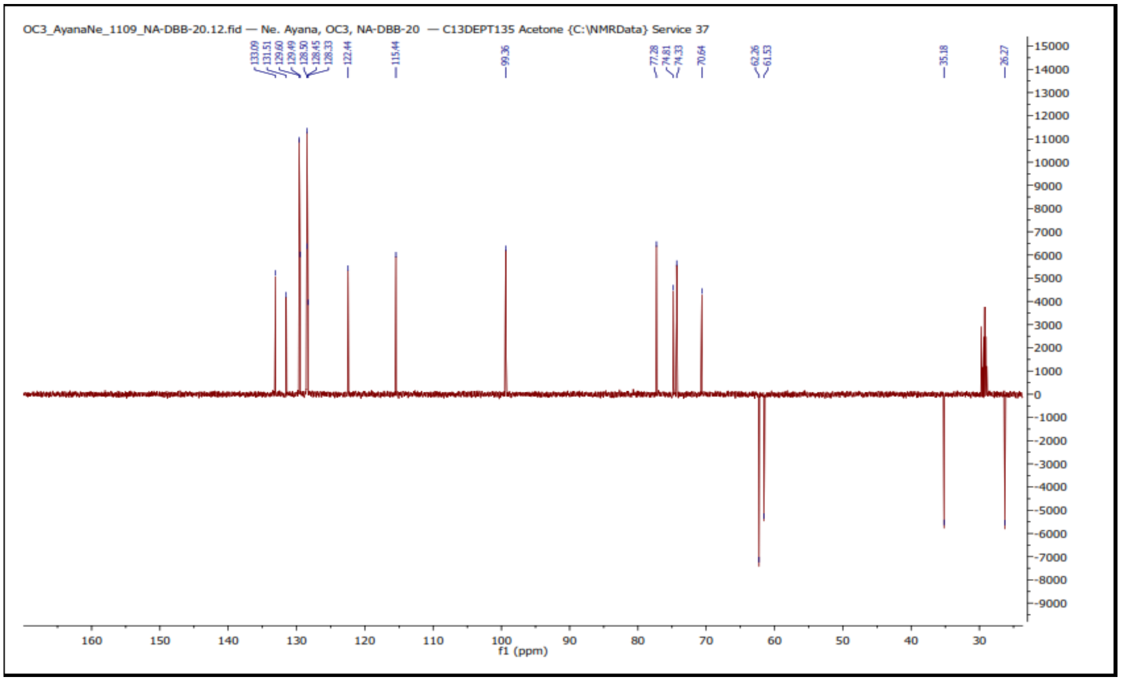

Figure S3. DEPT-135 spectrum (125 MHz, acetone-*d*6) of DBB-20/ Tremulacin (1)

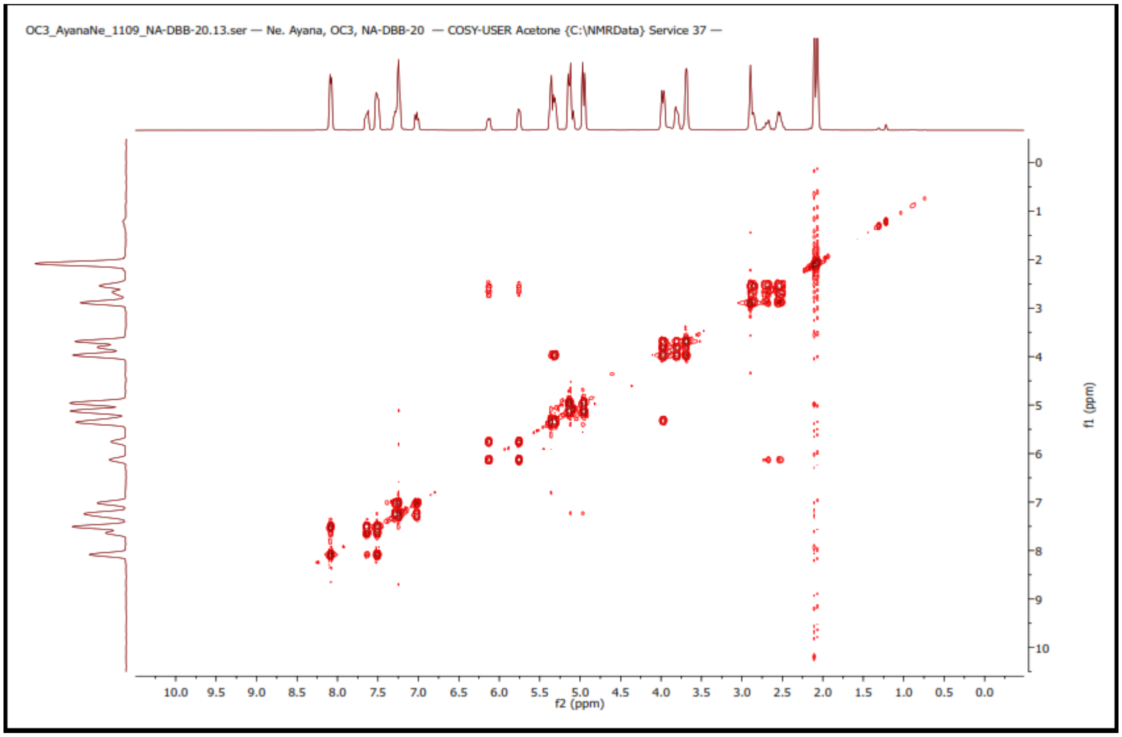

Figure S4. COSY spectrum (500 MHz, acetone-*d*6) of-DBB-20 / Tremulacin (1)

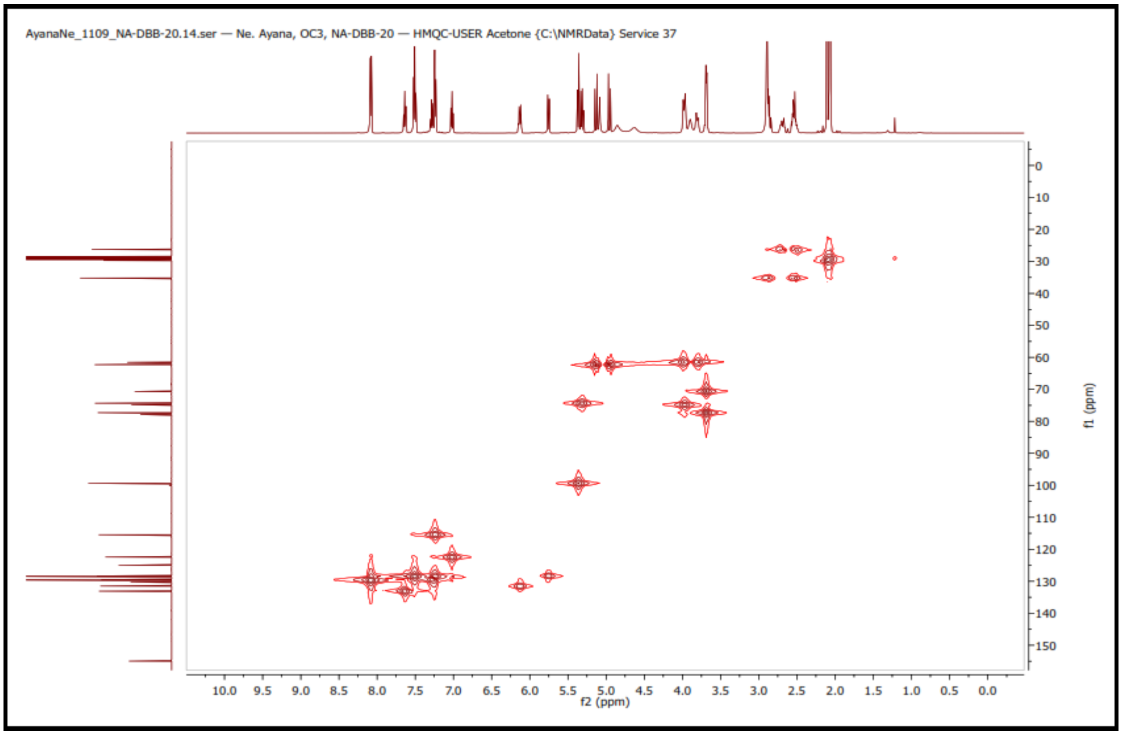

Figure S5. HMQC spectrum(500 MHz, 125 MHz acetone-*d*6) of DBB-20 / Tremulacin (1)

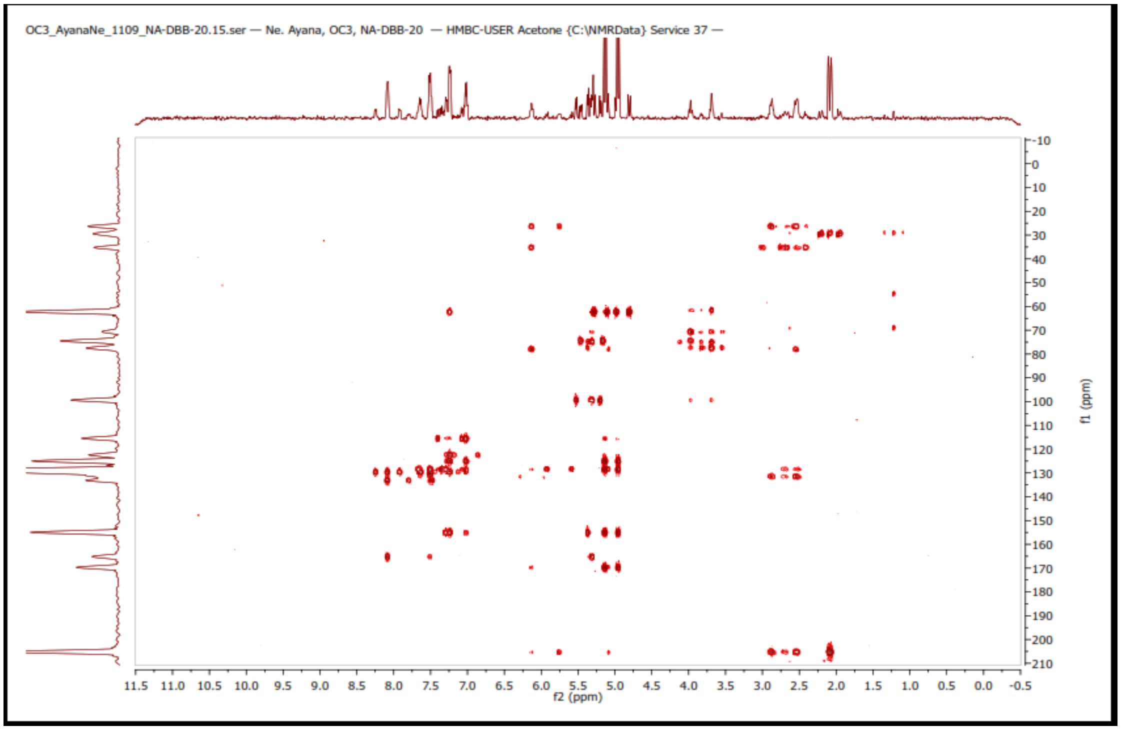

Figure S6. HMBC spectrum (500 MHz, 125 MHz acetone-*d*6) of DBB-20 / Tremulacin (1)

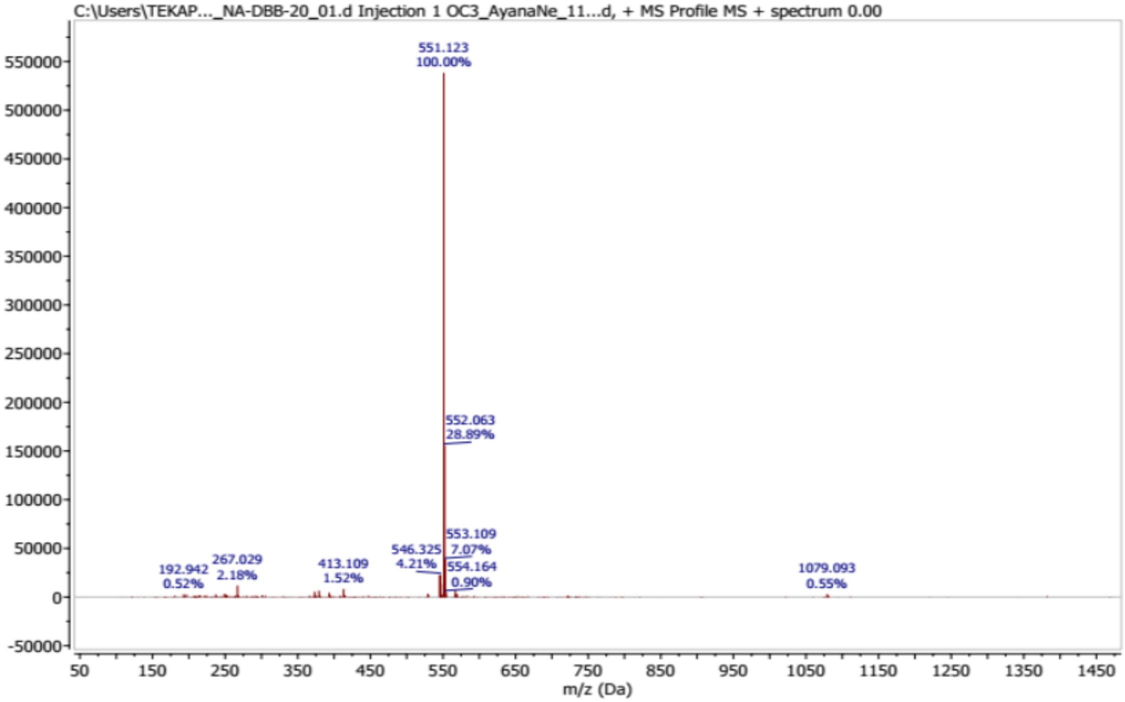

Figure S7: ESI-MS measurement or results of DBB-20/ Tremulacin (1)

Spectroscopic data for Compound 2

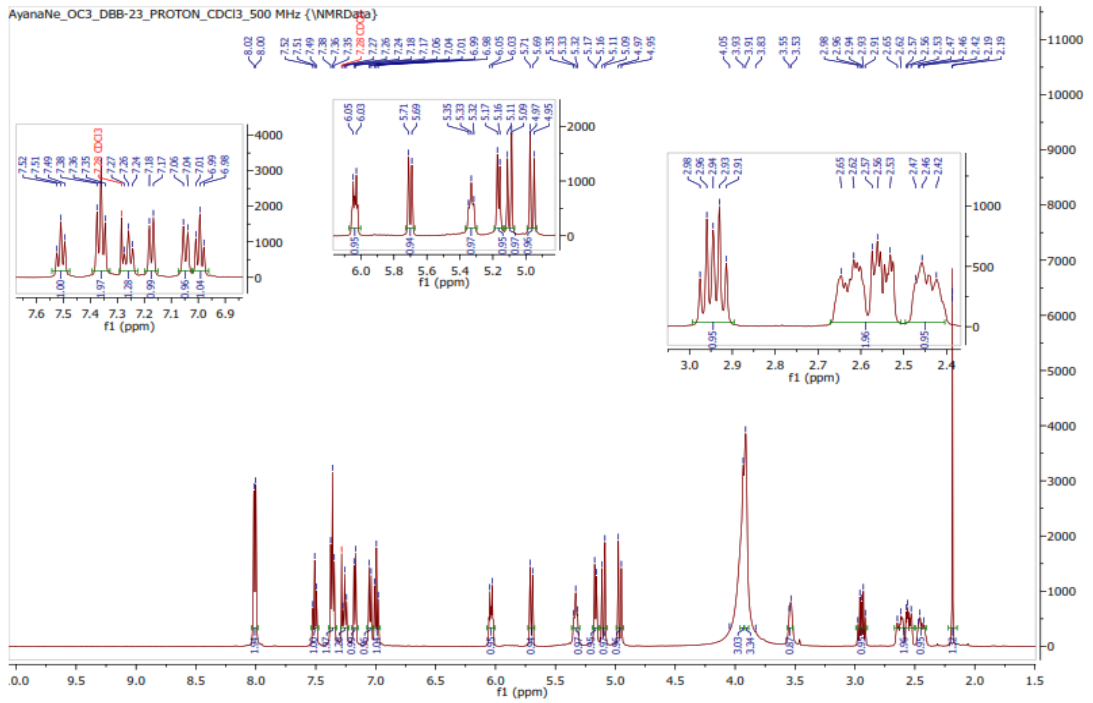

Figure S8. ^{1}H NMR spectrum (500 MHz, chloroform-*d*) DBB-23/ Cochinchiside A (2).

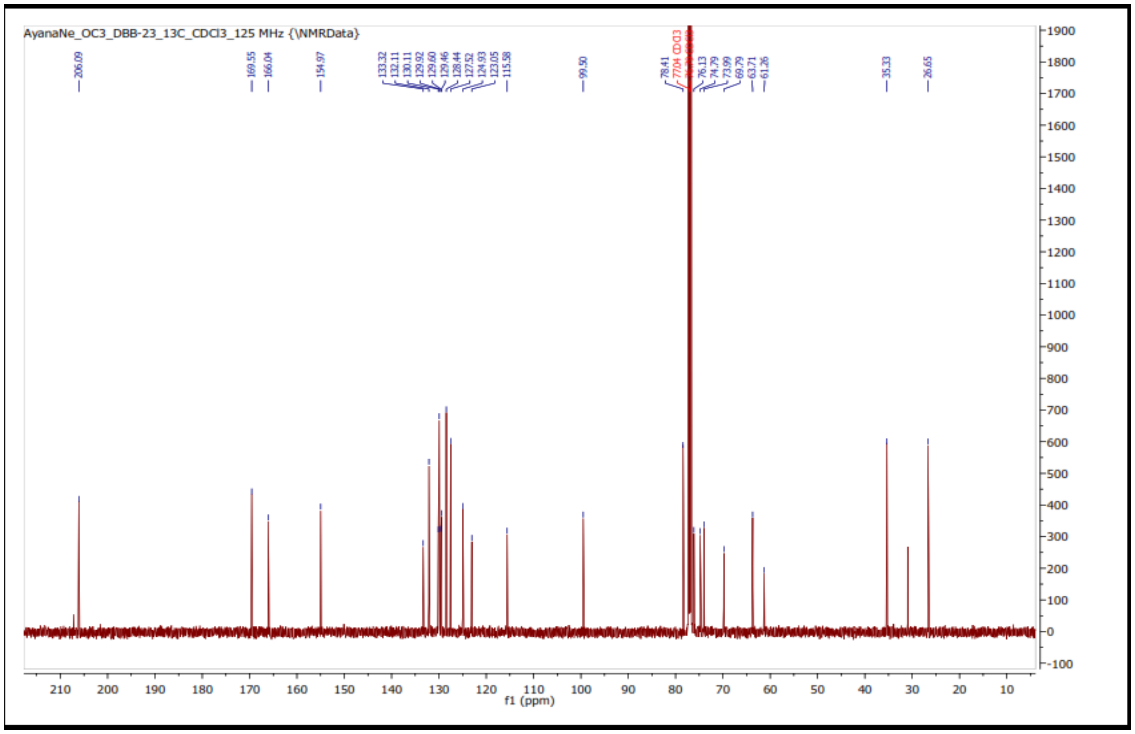

Figure S9. ^{13}C NMR spectrum(125 MHz, chloroform-*d*) of DBB-23/ Cochinchiside A (2).

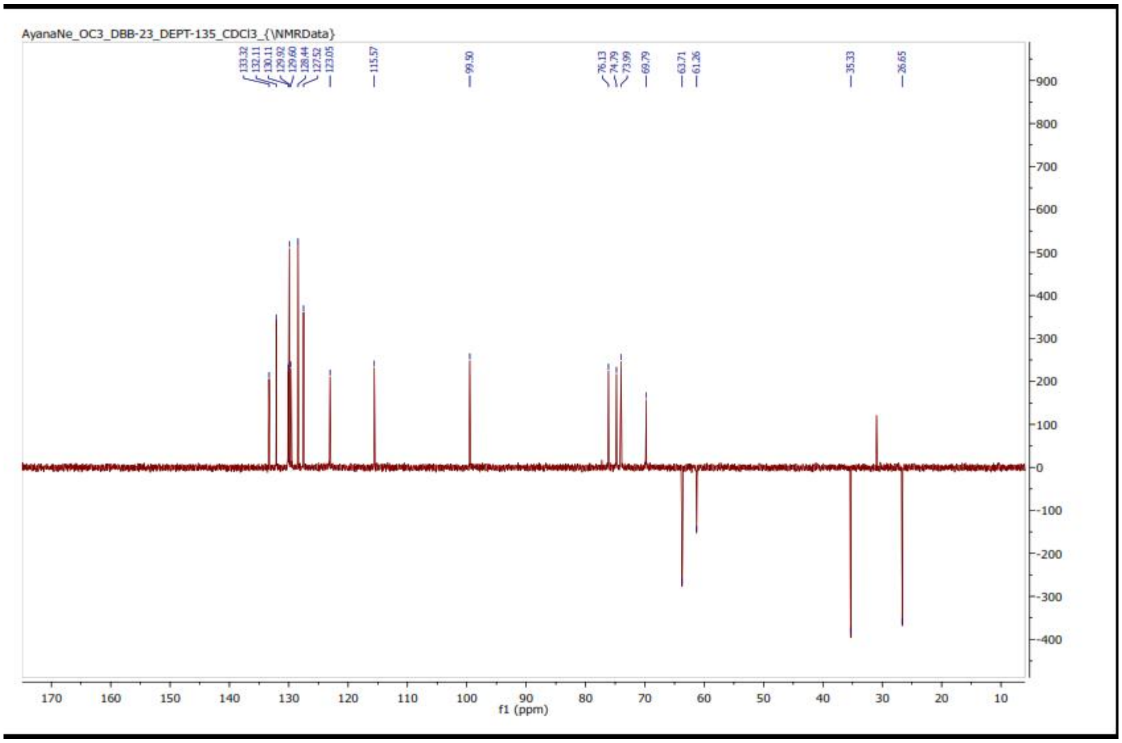

Figure S10. DEPT-135 spectrum (125 MHz, chloroform-*d*) of DBB-23/ Cochinchiside A (2)

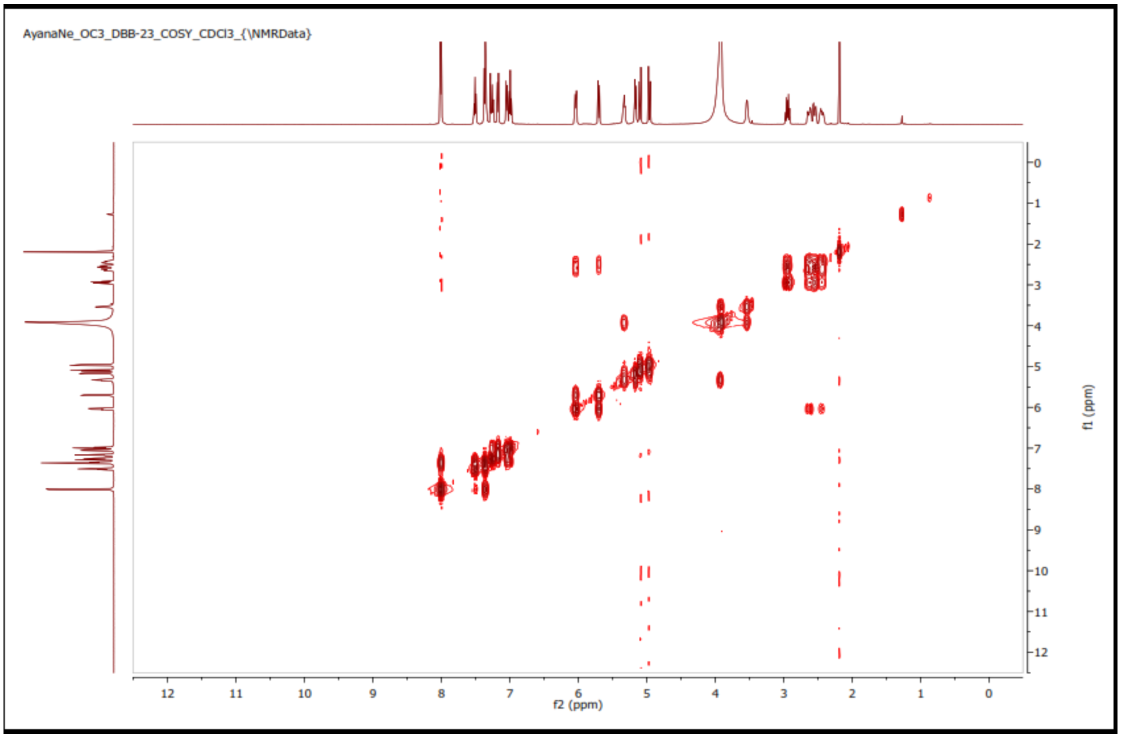

Figure S11. COSY spectrum (500 MHz, chloroform-*d*) of-DBB-23/ Cochinchiside A (2)

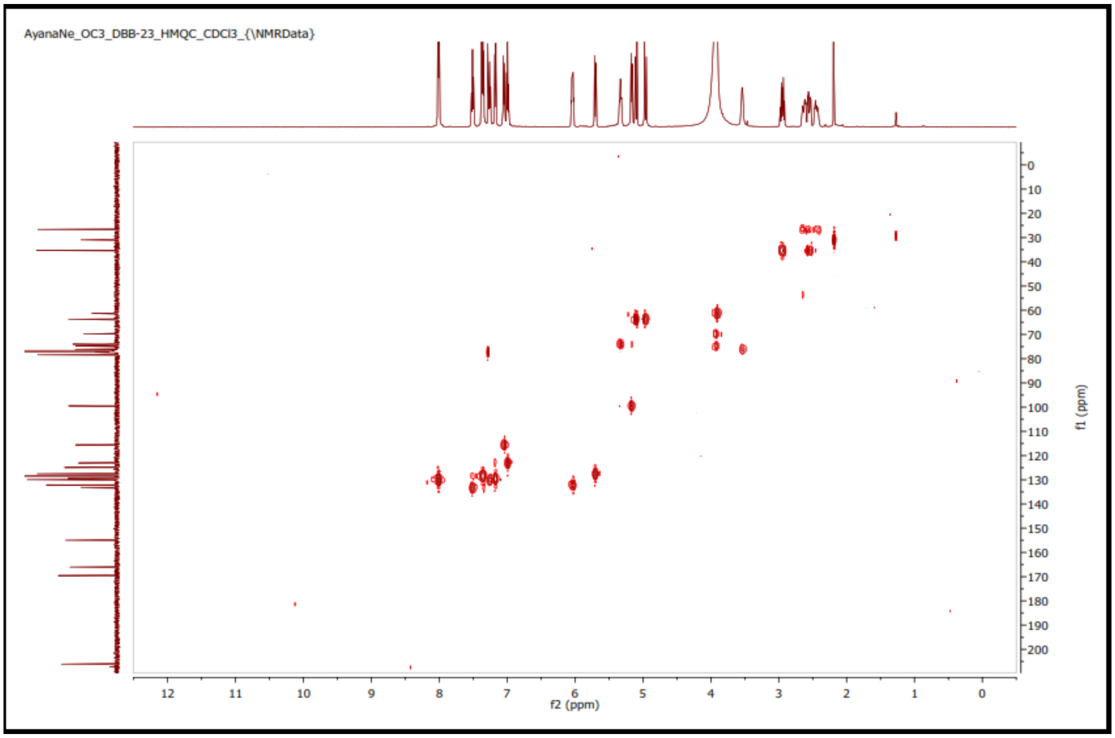

Figure S12. HMQC spectrum (500 MHz, 125 MHz chloroform-*d*) of DBB-23 Cochinchiside A (2)

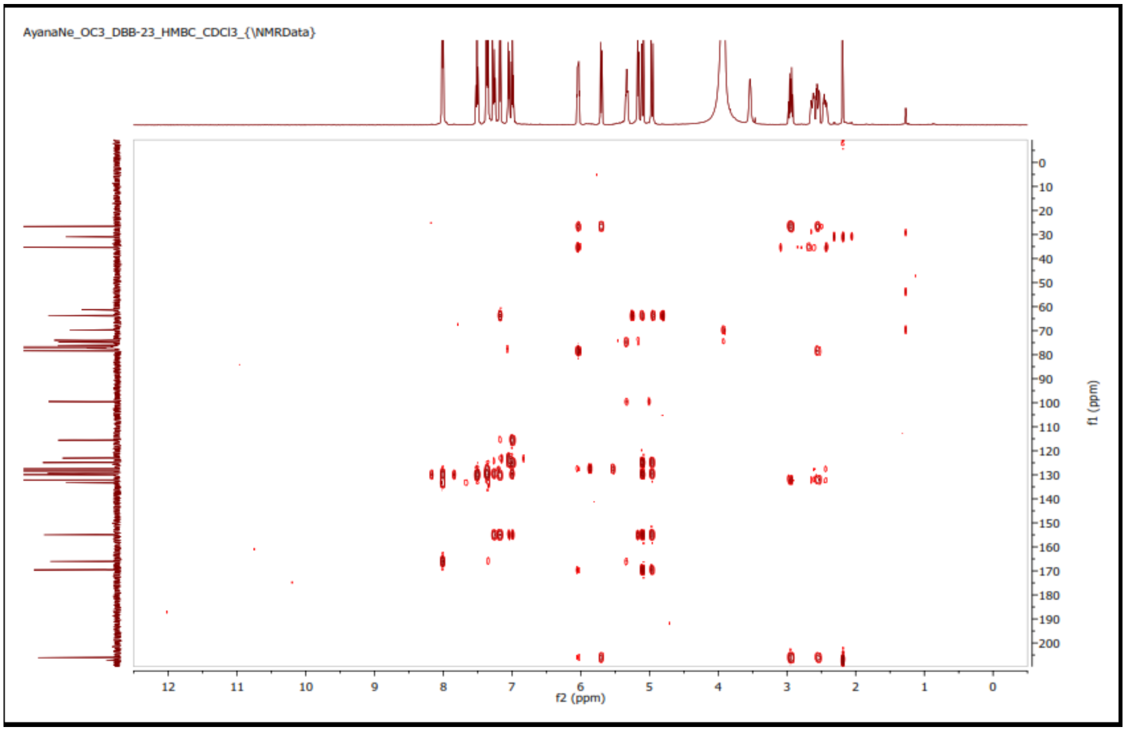

Figure S13. HMBC spectrum (500 MHz, 125 MHz chloroform-*d*) of DBB-23 Cochinchiside A (2)

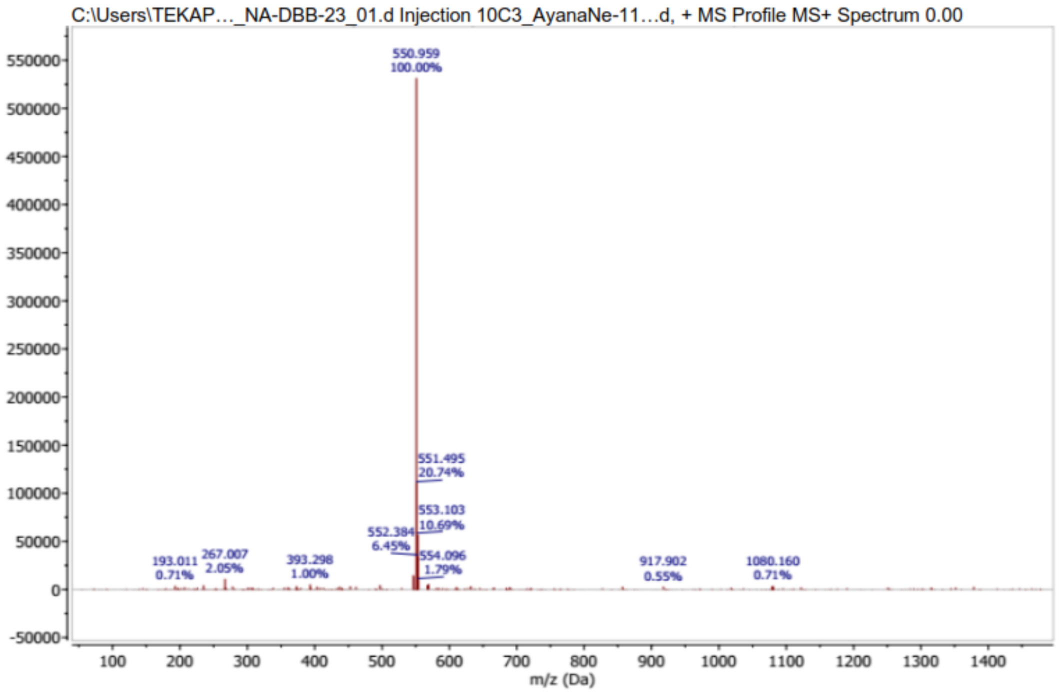

Figure S14: ESI-MS measurement or results of DBB-23/ Cochinchiside A (2)

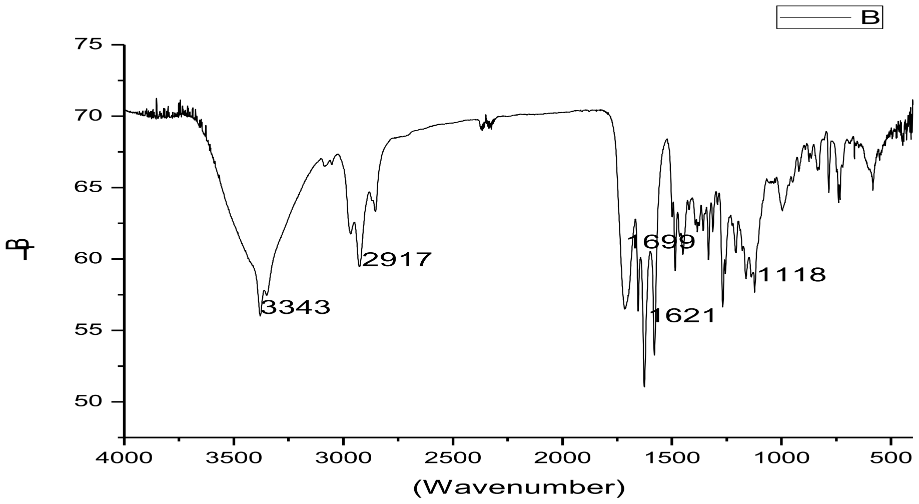

Figure S15: FT-IR spectrum of DBB-23/ Cochinchiside A (2)

Spectroscopic data for Compound 3

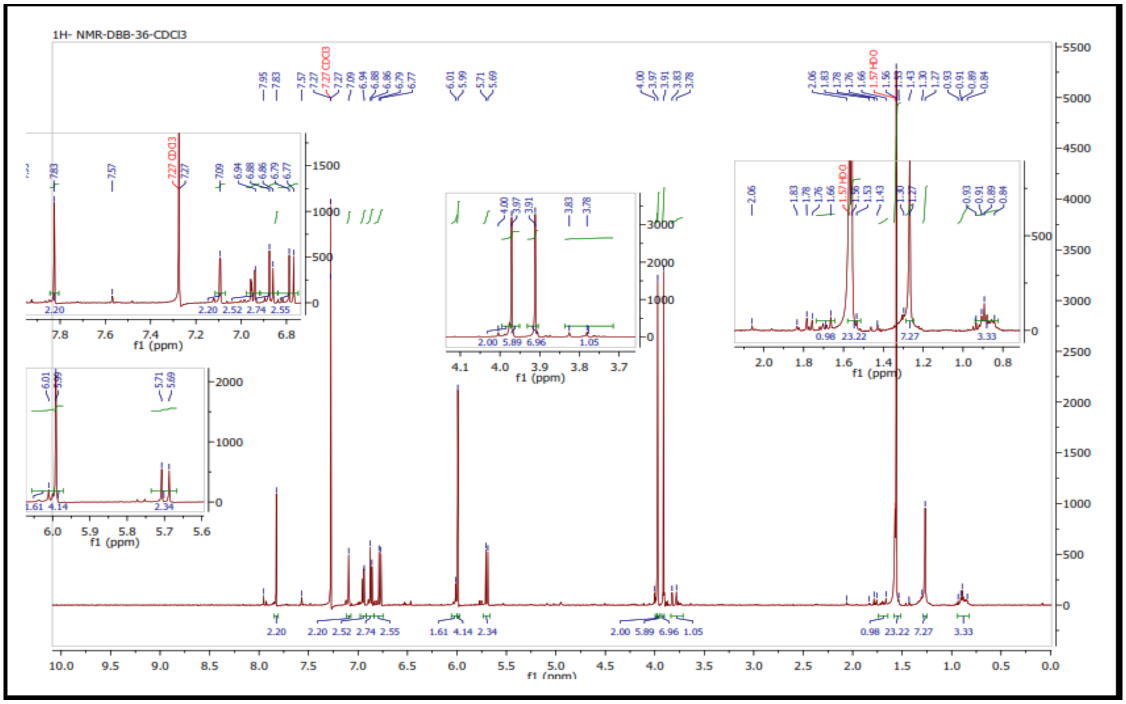

Figure S16. ^{1}H NMR spectrum (400 MHz, chloroform-*d*) of DBB-36/5-methoxy durmillone (3)

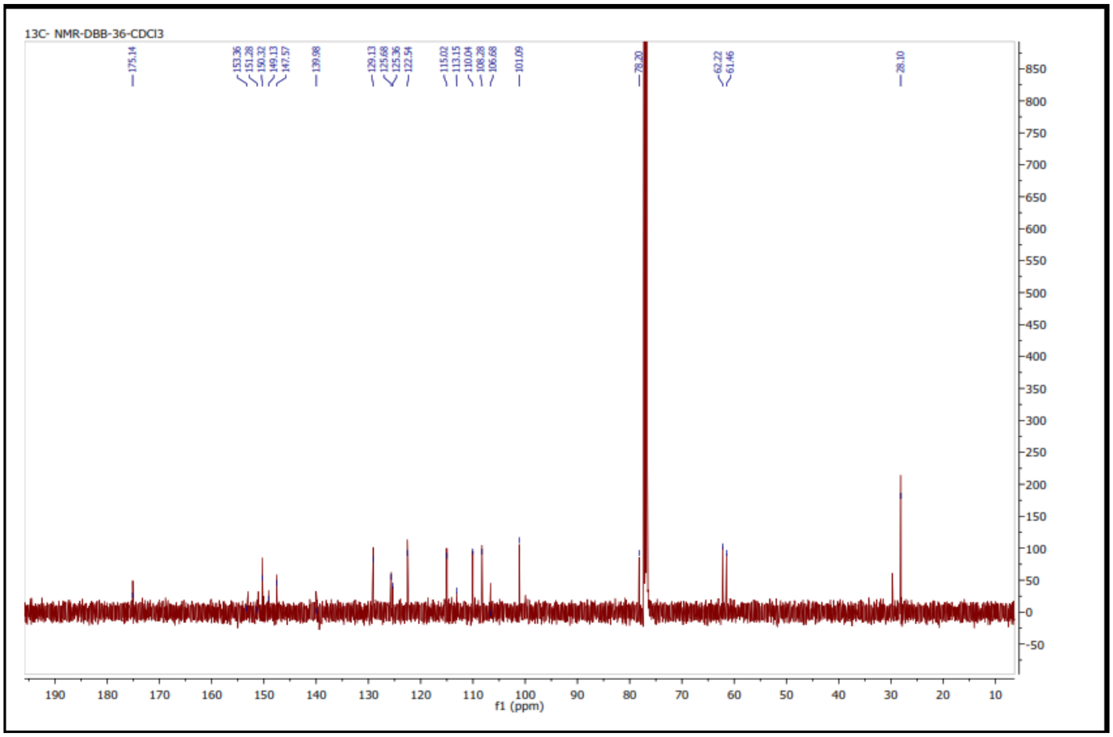

Figure S17. ^{13}C NMR spectrum (125 MHz, chloroform-d) of DBB-36/ 5-methoxy durmillone (3)

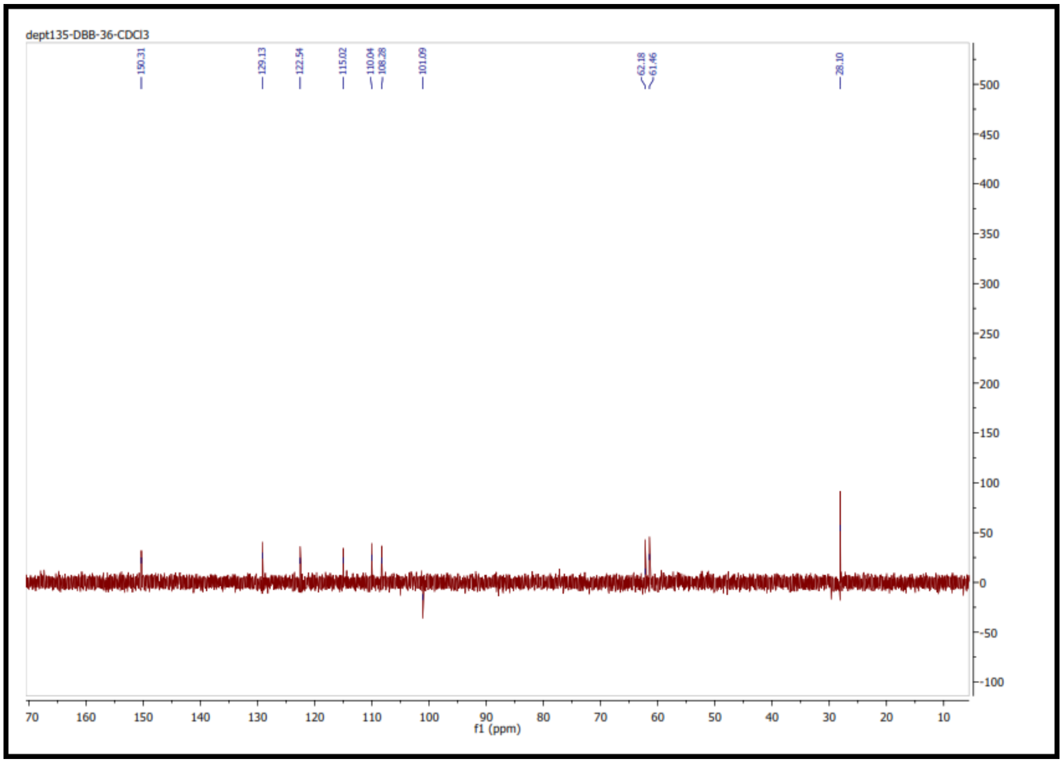

Figure S18 . DEPT-135 spectrum (125 MHz, chloroform-*d*) of DBB-36/ 5-methoxy durmillone (3)

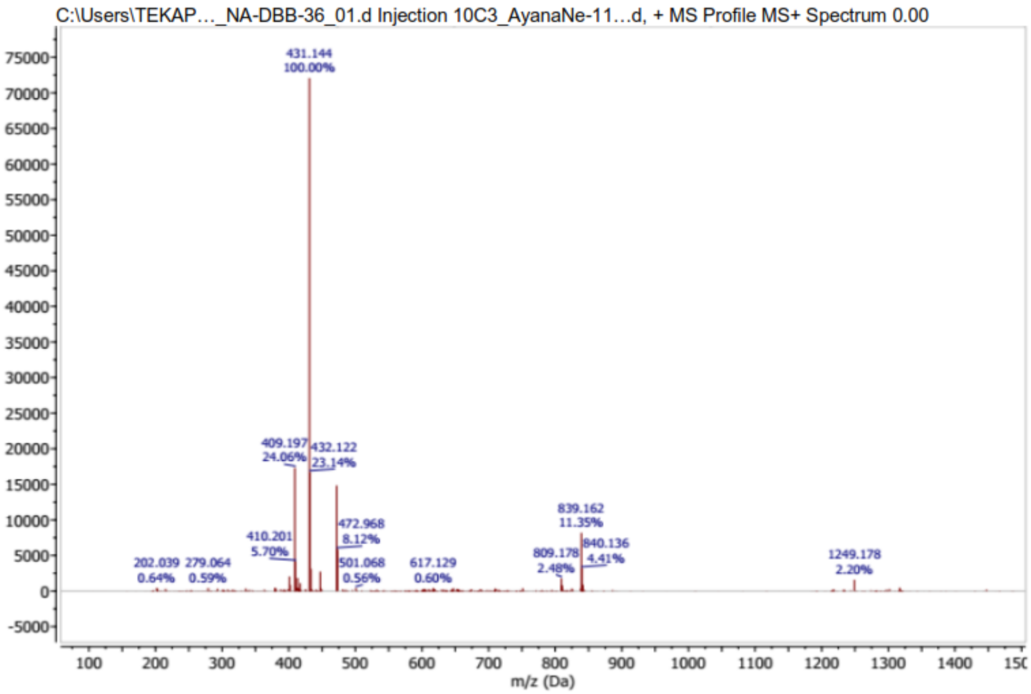

Figure S19: ESI-MS measurement or results of DBB-36/ 5-methoxy durmillone (3)

Spectroscopic data for Compound 4

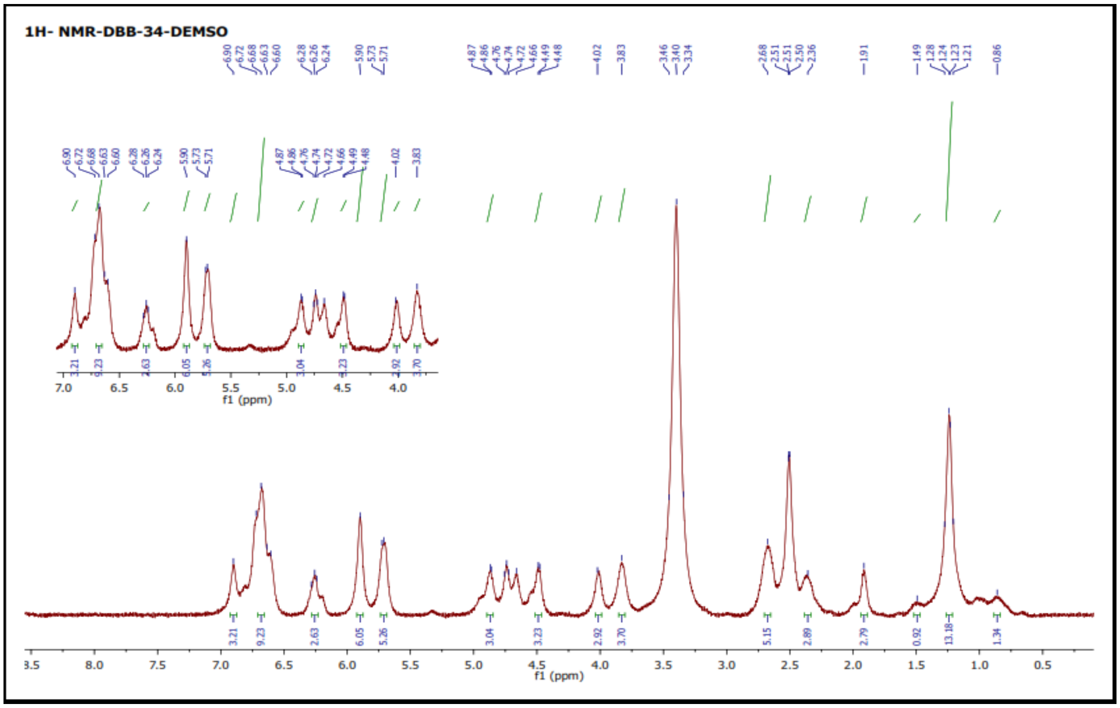

Figure S20. ^{1}H NMR spectrum(400 MHz, Dmso) of DBB-34/catechin-7-O-α -L-rhamnopyranoside (4)

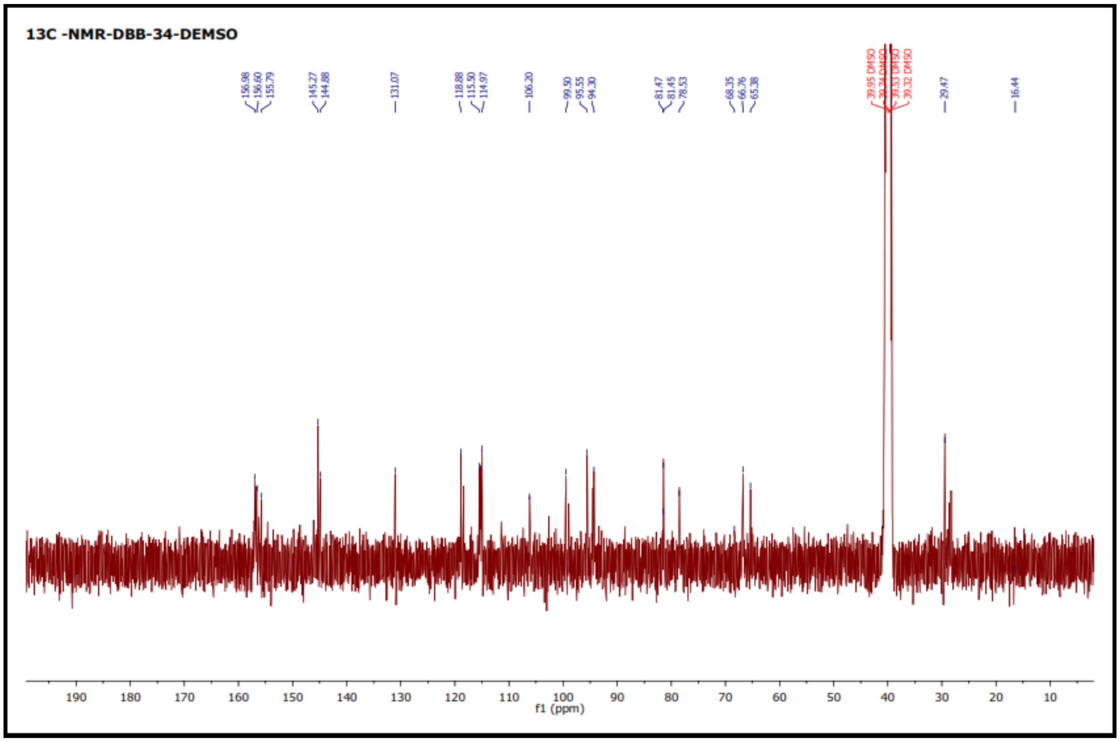

Figure S21. ^{13}C NMR spectrum (125 MHz, Dmso) of DBB-34 catechin-7-O-α -L-rhamnopyranoside (4)

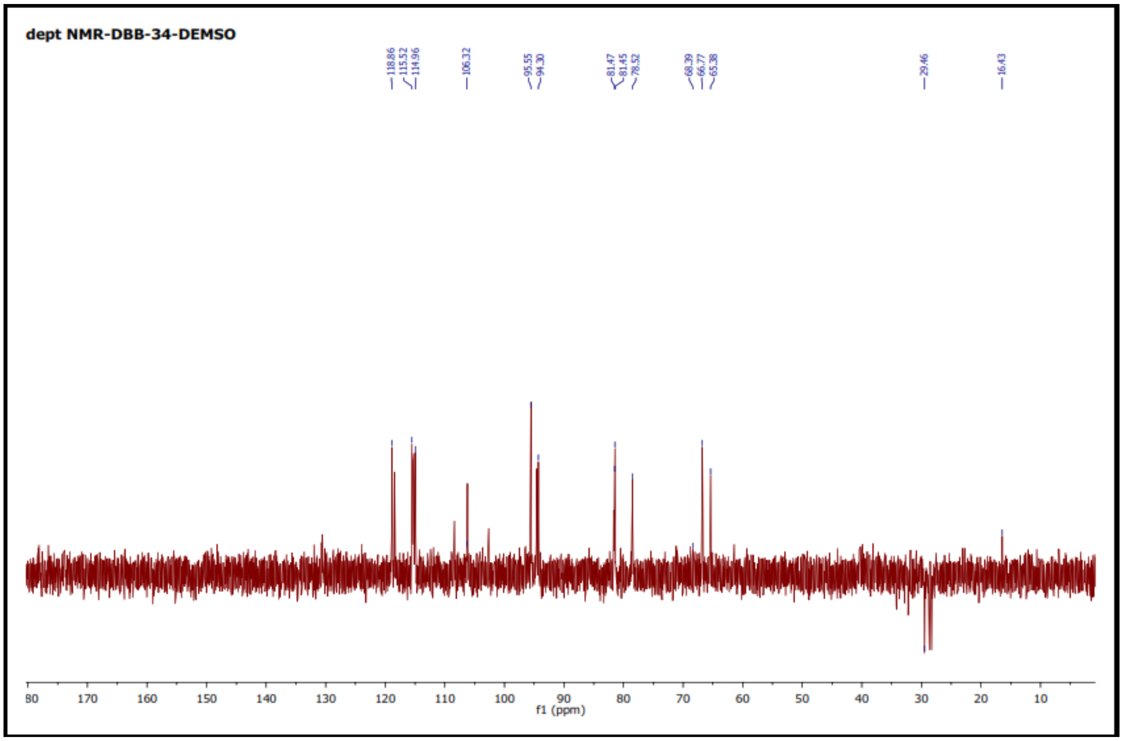

Figure S22. DEPT-135 spectrum (125 MHz, Dmso) of DBB-34/ Catechin-7-O-α -L-rhamnopyranoside (4)

Spectroscopic data for Compound 5

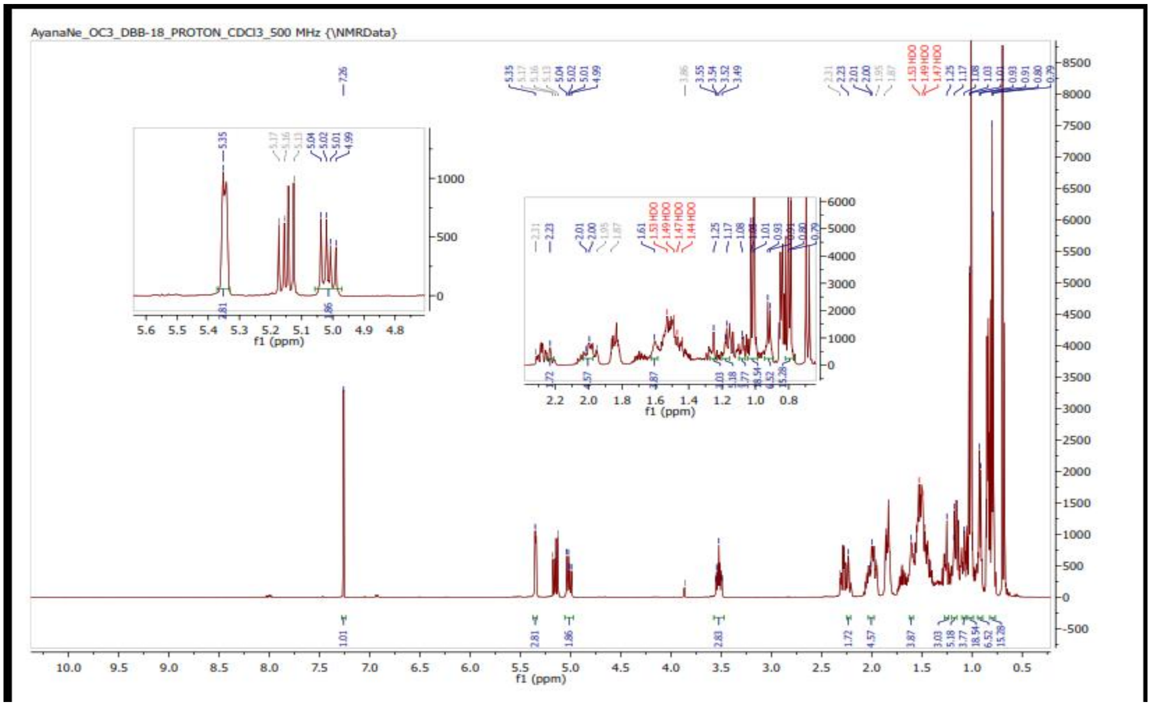

Figure S23. ^{1}H NMR spectrum(500 MHz, chloroform-*d*) of DBB-18/Stigmasterol(5)

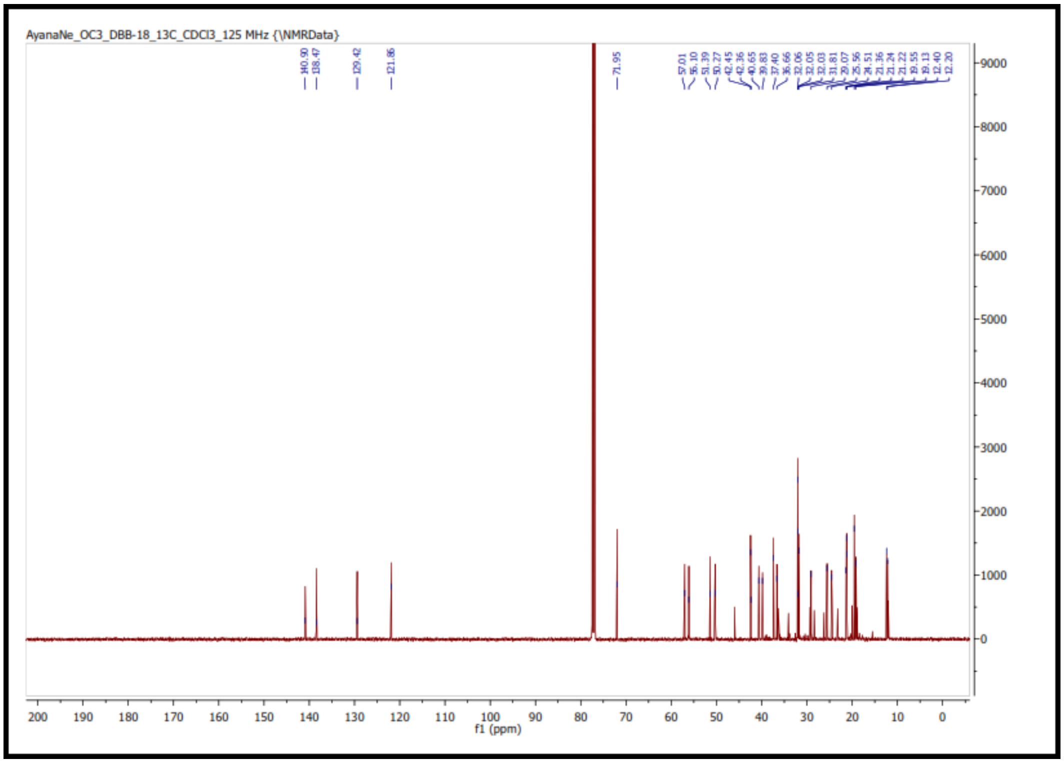

Figure S24. ^{13}C NMR spectrum(125 MHz, chloroform-*d*) of DBB -18/Stigmasterol(5)

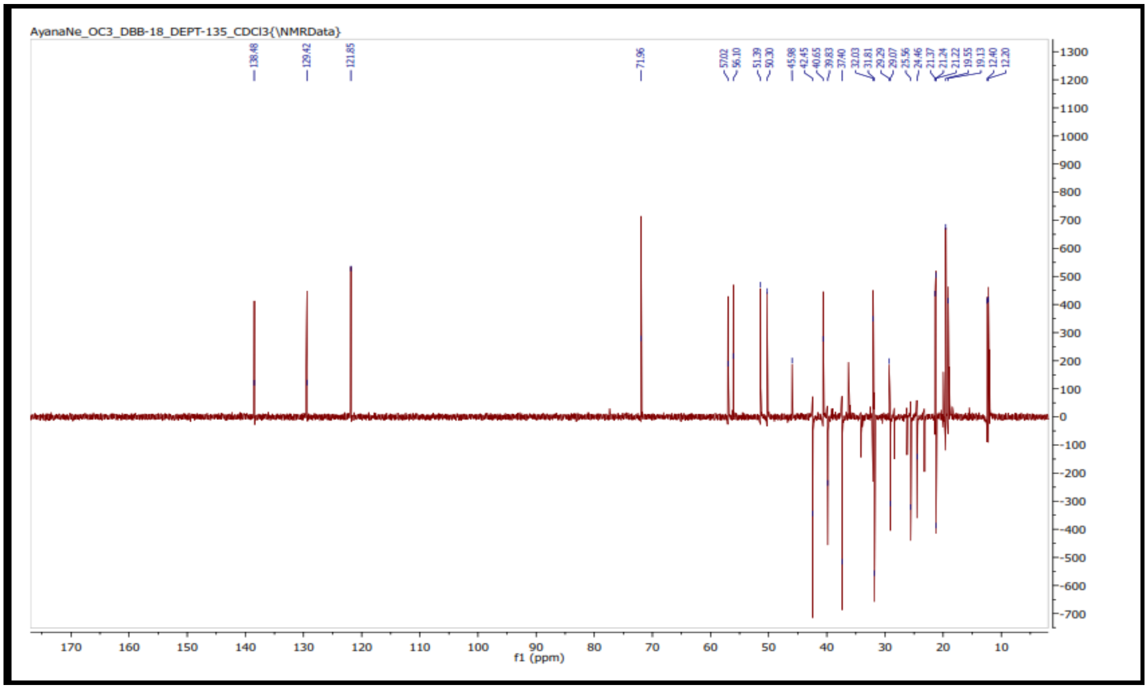

Figure S25. DEPT-135 spectrum(125 MHz, chloroform-*d*) of DBB -18/Stigmasterol (5)

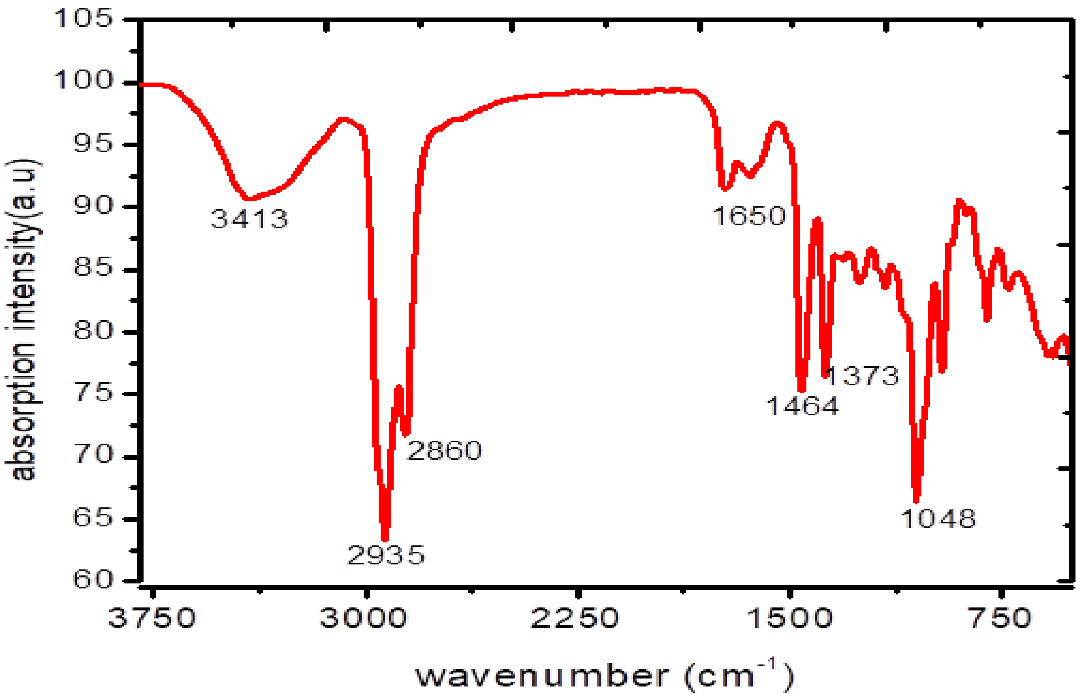

Figure S26: FT-IR spectrum of DBB -18/Stigmasterol (5)

Table S1. Molecular docking results of compounds **1**, **3**, and ciprofloxacin against Pyruvate kinase of S. aureus (PDB ID: 3T07) (Binding Affinity in kcal/mol)

Compounds	Binding Affinity	H-bond	Residual interactions	
			Hydrophobic	Van der Waals
**1** $C_{27}H_{28}O_{11}$	− 8.0	Lys342 (Dist.2.25672 Å), Lys342 (Dist.2.52025 Å), Lys349 (Dist.2.31698 Å), Asp346 (Dist.2.9578 Å)	Hydrophobic-Alkyl -Lys-342 (Dist.3.4.57773 Å), Hydrophobic-Pi-Alkyl-Lys-342 (Dist.3.5.42483 Å), Hydrophobic-Pi-Alkyl -Lys-342 (Dist.3.4.09151 Å), Hydrophobic-Pi-Alkyl-Leu-343 (Dist.3.5.11034 Å)	Arg-264, Tyr-302, Asp-303, Gln-338, Asp-339, Leu-343, Ser-345, Lys-349
**3** $C_{23}H_{20}O_7$	− 9.0	Ser-362, Asn-369 (Dist. 2.71417 Å), Asn-369 (Dist. 2.61409 Å)	Hydrophobic-Pi-Sigma- Ile-361 (Dist. 3.82656 Å), Ile-361 (Dist. 3.60133 Å), His-365Hydrophobic-Pi-Pi-Stacked-His365 (Dist.5.92423 Å), His-365 (Dist. 4.79808 Å), His-365 (Dist. 4.58193 Å)	Thr-348, Thr-353, Asn-369, Thr-366, Ser-362
Ciprofloxacin ($C_{17}H_{18}FN_3O_3$)	− 8.3	Ser-362 (Dist. 2.76717 Å), Ser-362 (Dist. 1.76537 Å), Ser-362 (Dist. 3.31994 Å), Thr-366, Asn-369, Ala-358	Hydrophobic-Pi-Pi-Stacked- His-365	Ile-361, Thr-348, His-365, Asn-369

Table S2. Molecular docking results of compounds **1**, **3,** and ciprofloxacin against *S. epidermidis* FtsZ (PDB ID: 4M8I) (Binding Affinity in kcal/mol)

Compounds	Binding Affinity	H-bonding	Residual interactions	
			Hydrophobic, Electrostatic, and others	Van der Waals
**1** $C_{27}H_{28}O_{11}$	-9.7	Gly-21 (Dist. 2.77217 Å), Gly-22 (Dist. 2.01581 Å), Gly-107 (Dist. 2.11888 Å), Gly-108 (Dist. 2.14157 Å), Thr-109 (Dist. 1.82979 Å), Arg-143 (Dist. 2.79671 Å), Arg-143 (Dist. 1.76664 Å) Asn- 166 (Dist. 2.6524 Å), Met-105 (Dist. 2.69428 Å), Glu-139 (Dist. 2.92938 Å), Gly-107 (Dist. 3.53693 Å), UNL1:C (Dist. 3.69667 Å)	Hydrophobic-Pi-Pi Stacked-Phe-183 (Dist. 3.85018 Å)	Val-19, Gly-20, Gly-23, Asn-25, Arg-29, Asn-44, Asp-46, Gly-70, Ala-71, Gly-72, Ala-73, Asn-74, Ala-103, Gly-106, Gly-110, Thr-133
**3** $C_{23}H_{20}O_{7}$	-8.7	Asn-25 (Dist. 2.8773 Å), Asn-25 (Dist. 2.62015 Å)	Electrostatic-Pi-Cation- Arg-143	Gly-21,Phe-183, Phe-136, Asn-166, Arg-29, Thr-133, Gly-104, Gly-22, Gly-107
Ciprofloxacin ($C_{17}H_{18}FN_{3}O_{3}$)	-8.0	Thr-45, Ala-71, Gly-108, Thr-109 (Dist. 2.11448 Å), Thr-109 (Dist. 2.28525 Å), Gly-110, Asn-44, Gly-70, Glu-139, Gly-22	Halogen (Fluorine) Acceptor- Gly-104 Halogen (Fluorine) Acceptor- Met-105	Phe-136, Phe-183, Asn-166, Gly-107, Thr-111, Ala-73, Gly-72, Gly-20, Asp-46, Gly-21, Asn-25

Table S3. Molecular docking results of compounds **1**, **3** and ciprofloxacin against *K. pneumoniae* Topoisomerase IV (ParE-ParC) in complex with DNA (PDB ID: 7LHZ) (Binding Affinity in kcal/mol)

Compounds	Binding Affinity	H-bond	Residual interactions	
			Hydrophobic and others	Van der Waals
**1** $C_{27}H_{28}O_{11}$	-8.0	Phe-1147 (Dist. 2.05181Å), Asp-493 (Dist. 2.23686 Å), Lys-565 (Dist. 2.79239 Å), UNL1: H-UNL1: O (Dist. 2.73799 Å)	Electrostatic-Pi-Anion-Asp-493 (Dist.3.73885 Å), Hydrophobic-Pi-Pi T-shaped-UNL1 (Dist.4.89606 Å), Hydrophobic-Pi-Alkyl-Tyr-1120 (Dist.5.47968 Å)	Dte-11, Asp-491, Ala-492, Ser-494, Leu-527, Tyr-528, Arg-563, Phe-564, Gly-566, Tyr-1024, Pro-1076, His-1077, Arg-1119, Asp-1148
**3** $C_{23}H_{20}O_{7}$	-8.4	Arg-1372 (Dist. 2.59151 Å), Arg-1372 (Dist. 2.38503 Å), Arg-1372 (Dist. 2.61195 Å)	Hydrophobic-Pi-Sigma-Ile-1375, Ala-1416, Hydrophobic-Pi-Alkyl-Ile-1375, Ala-1416, Leu-1379	Thr-1412, Glu-1415, Glu-1419, Leu-1420, His-1424, Ile-1433
Ciprofloxacin ($C_{17}H_{18}FN_{3}O_{3}$)	- 6.7	Asn-1385,Lys-555, Gly-557, Glu-550	Halogen(Fluorine) Acceptor- Leu-549 Hydrophobic Alkyl- Val-	Ile-1409, Gly-1408, Thr-546, Pro-559, Lys-558, Leu-552,

	1381 Hydrophobic-Pi-Alkyl-Lys-553	Lys-556, Leu-1384, Phe-1407

Information/photo of Antibacterial activity

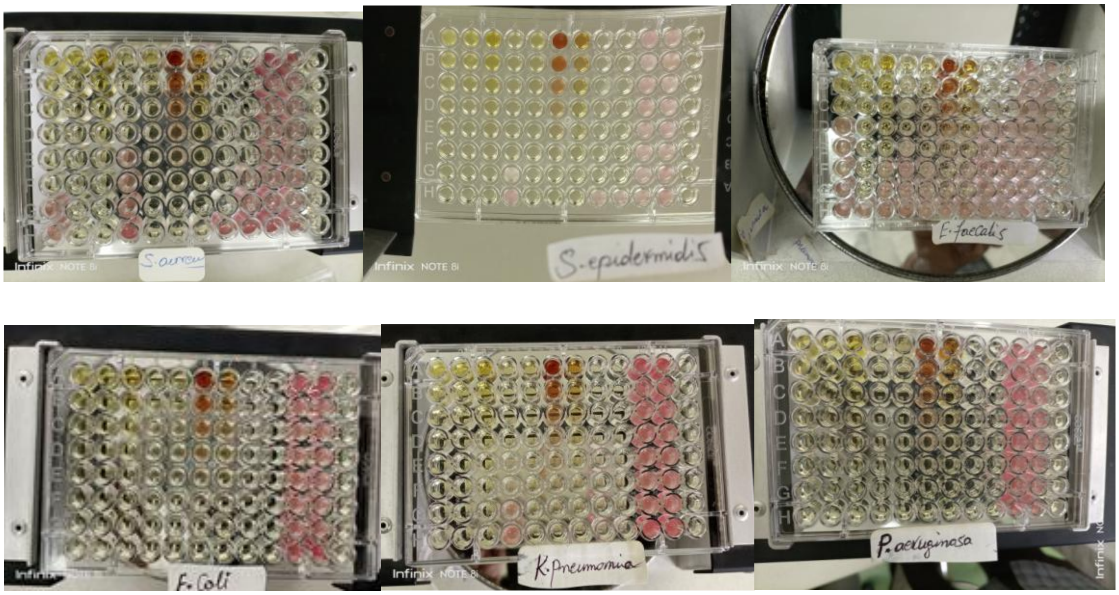

Keynotes: For the 96-microwell plate, starting with the first to seventh raw for sample 8th and 9th row for positive (Ciprofloxacin) control and 10th and 11th for negative and growth control, 12 for sterility control. The 4th raw was my target plant, *Dovyalis abyssinica*

Figure S27. Photo of Antibacterial activity by using the micro-broth dilution method MIC values of the crude extracts against tested microorganisms

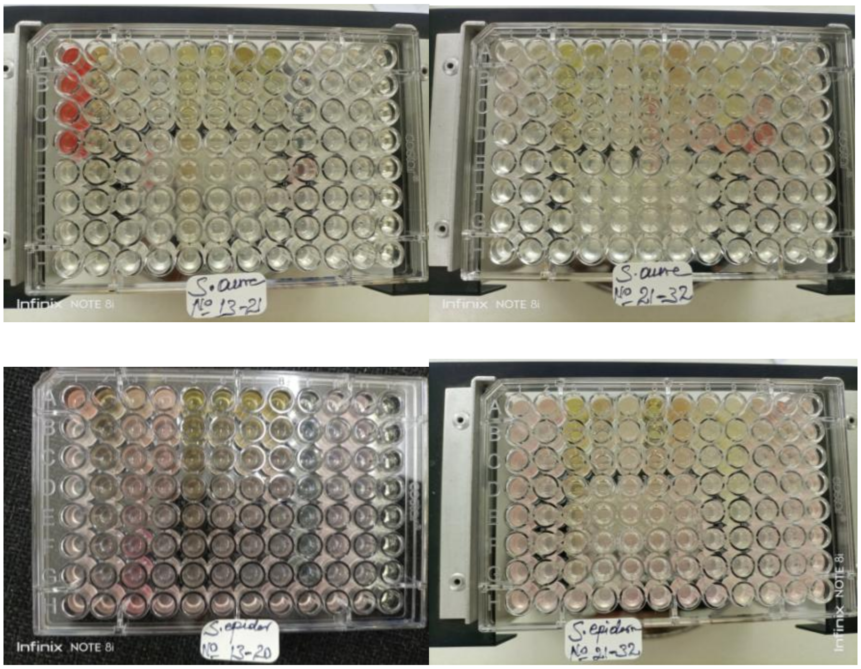

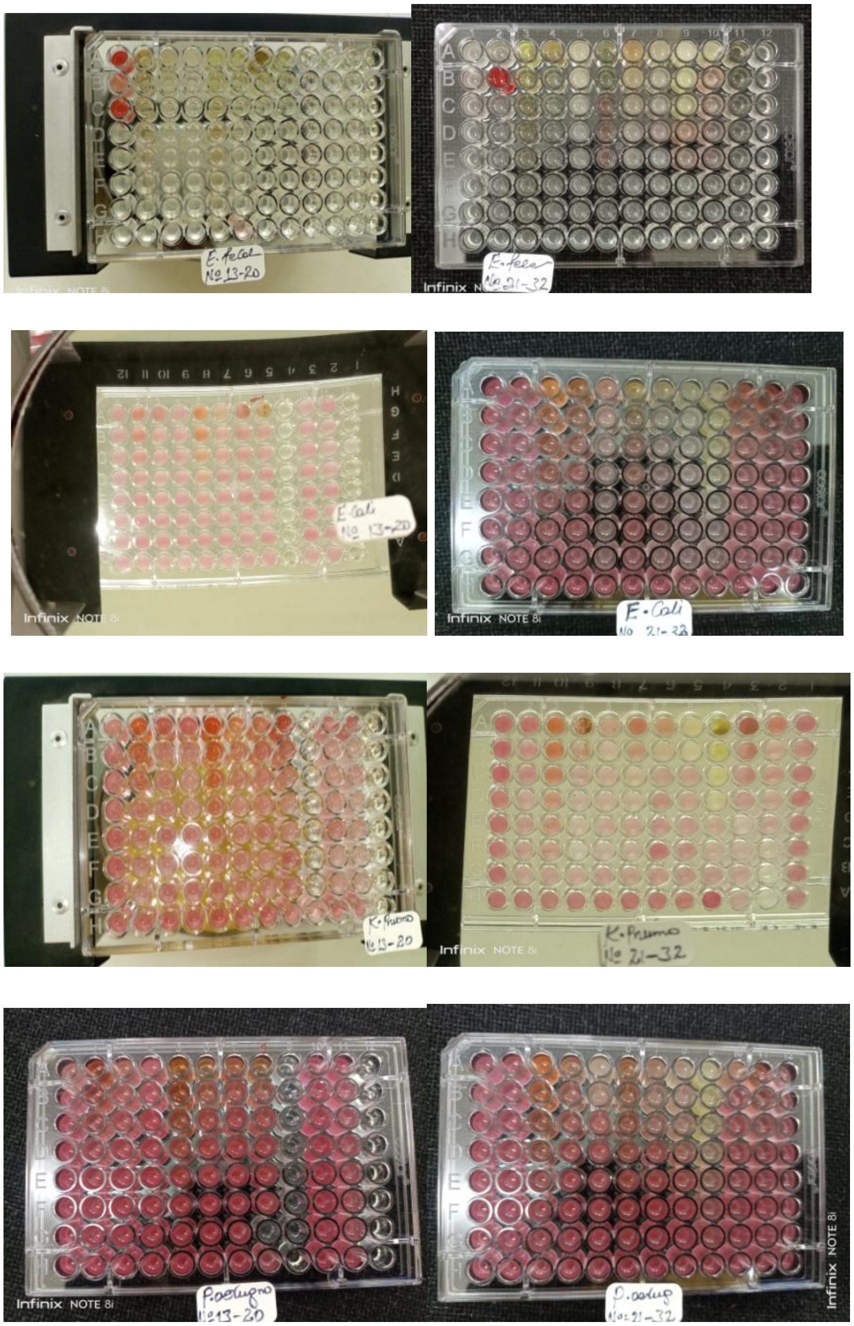

Keynotes: For the first 96-microwell plate, starting with the first raw for sample 13 and the eighth raw for sample 20, raw 9 for positive control (Ciprofloxacin), raw 10 and 11 for sterility and growth control, and raw 12 for negative control; for the second 96-microwell plate, beginning with the first raw for sample 21 and ending with the 12th raw for sample 32. The description includes raw 17 for sample dbb-36 (compound **3**), raw 18 for sample dbb-34 (compound **4**), raw 19 for sample dbb-23 (compound **2**), raw 20 for sample dbb-18 (compound **5**), and raw 23 for sample dbb-20 (compound **1**). The MIC values are expressed in mg/mL for crude extracts and isolated compounds, and μg/mL for positive controls.

Figure S28. Photo of Antibacterial activity by using the micro-broth dilution method MIC values of the isolated compounds against tested microorganisms
